# Supplementary material for: Graphitic phosphorus coordinated single Fe atoms for hydrogenative transformations
Source: Nat Commun. 2020 Aug 13;11:4074. doi: 10.1038/s41467-020-17903-0 (PMC7426939; doi:10.1038/s41467-020-17903-0)
Supplement: Supplementary file 1 — Supplementary Information [file 41467_2020_17903_MOESM1_ESM.pdf]

Supplementary Information

Graphitic phosphorus coordinated single Fe atoms for hydrogenative transformations

Long et al

Supplementary Figures

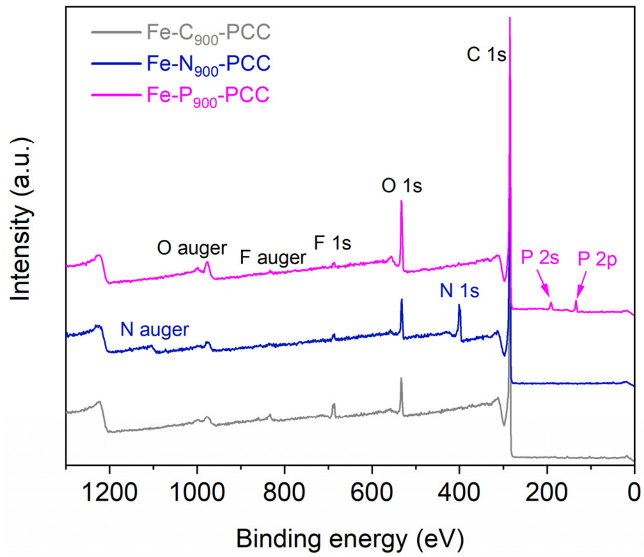

Supplementary Figure 1. XPS survey spectra of the Fe-C$_{900}$-PCC, Fe-N$_{900}$-PCC and Fe-P$_{900}$-PCC. The main elemental contributions are indicated.

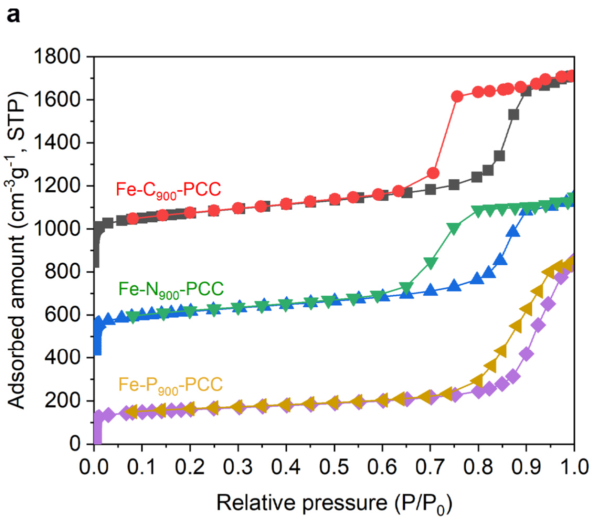

Supplementary Figure 2. Textural properties. N_2 adsorption-desorption isotherms (**a**) and pore size distribution curves (**b**) for Fe-C_{900}-PCC, Fe-N_{900}-PCC, and Fe-P_{900}-PCC.

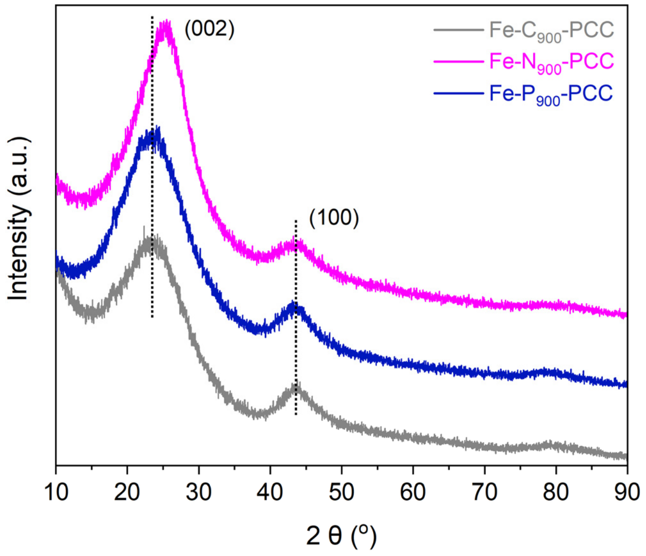

Supplementary Figure 3. XRD patterns of the Fe-C_{900}-PCC, Fe-N_{900}-PCC and Fe-P_{900}-PCC. The diffraction peaks (002, 100) are labelled in the spectra.

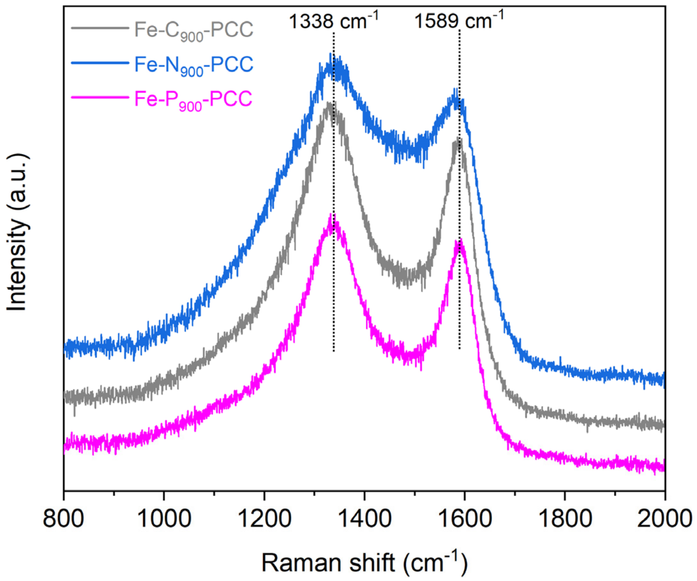

Supplementary Figure 4. Raman spectra of the Fe-C_{900}-PCC, Fe-N_{900}-PCC and Fe-P_{900}-PCC. For all of the samples, only D-band (1338 cm^{-1}) and G-band (1589 cm^{-1}) of carbon have been detected.

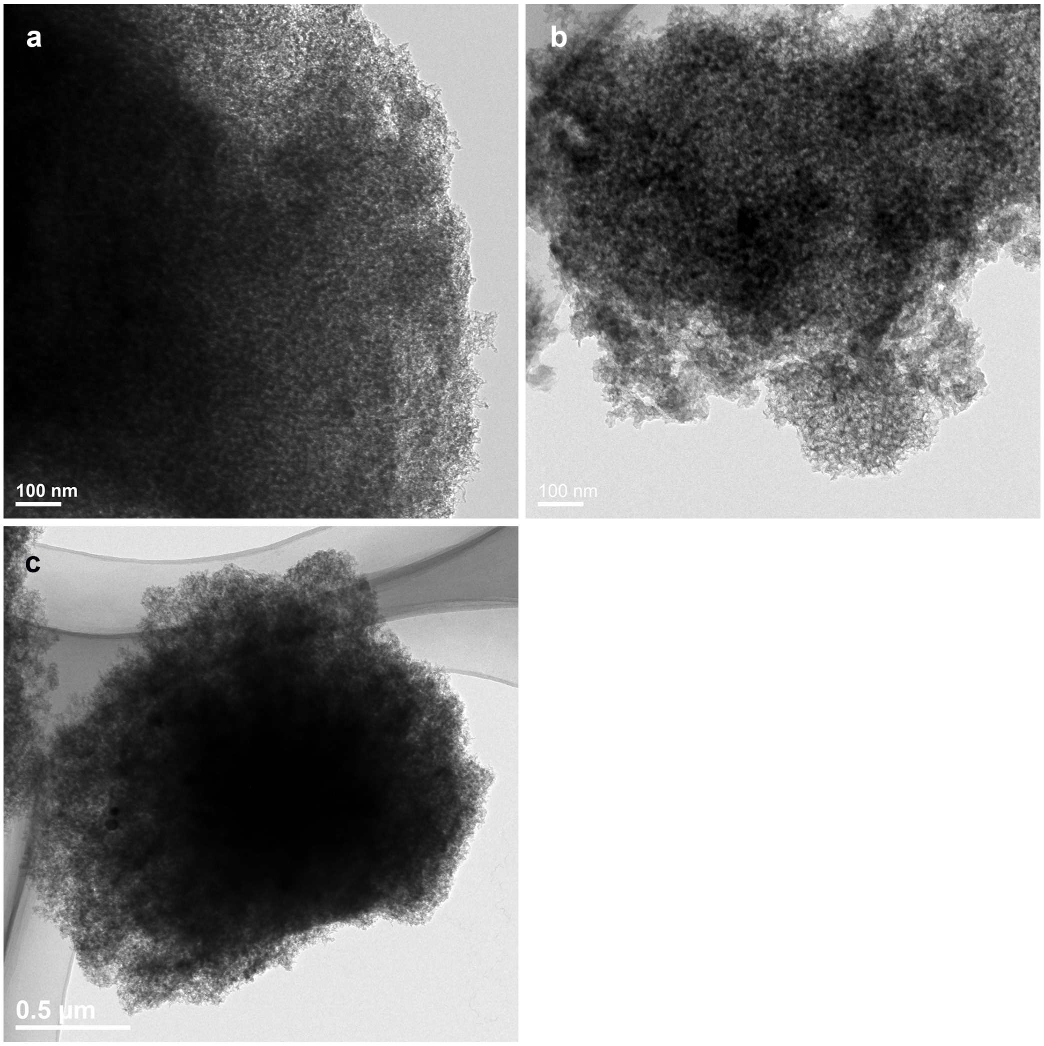

Supplementary Figure 5. Morphology characterization of the catalysts. TEM images of (**a**) Fe-C$_{900}$-PCC, (**b**) Fe-N$_{900}$-PCC and (**c**) Fe-P$_{900}$-PCC.

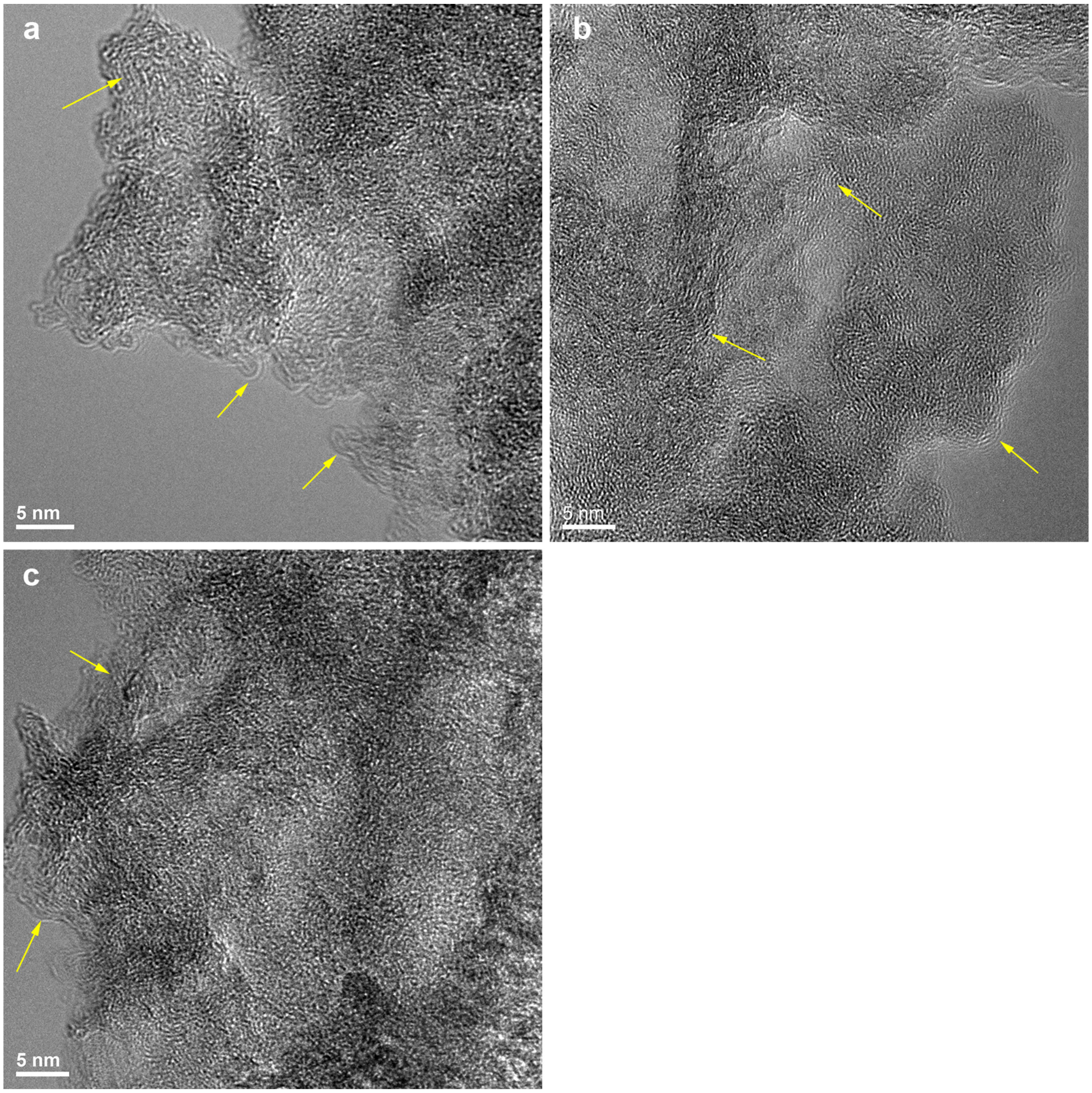

Supplementary Figure 6. HRTEM images of the catalysts. (a) Fe-C_{900}-PCC. **(b)** Fe-N_{900}-PCC. **(c)** Fe-P_{900}-PCC. Graphitic layers are highlighted by yellow arrows.

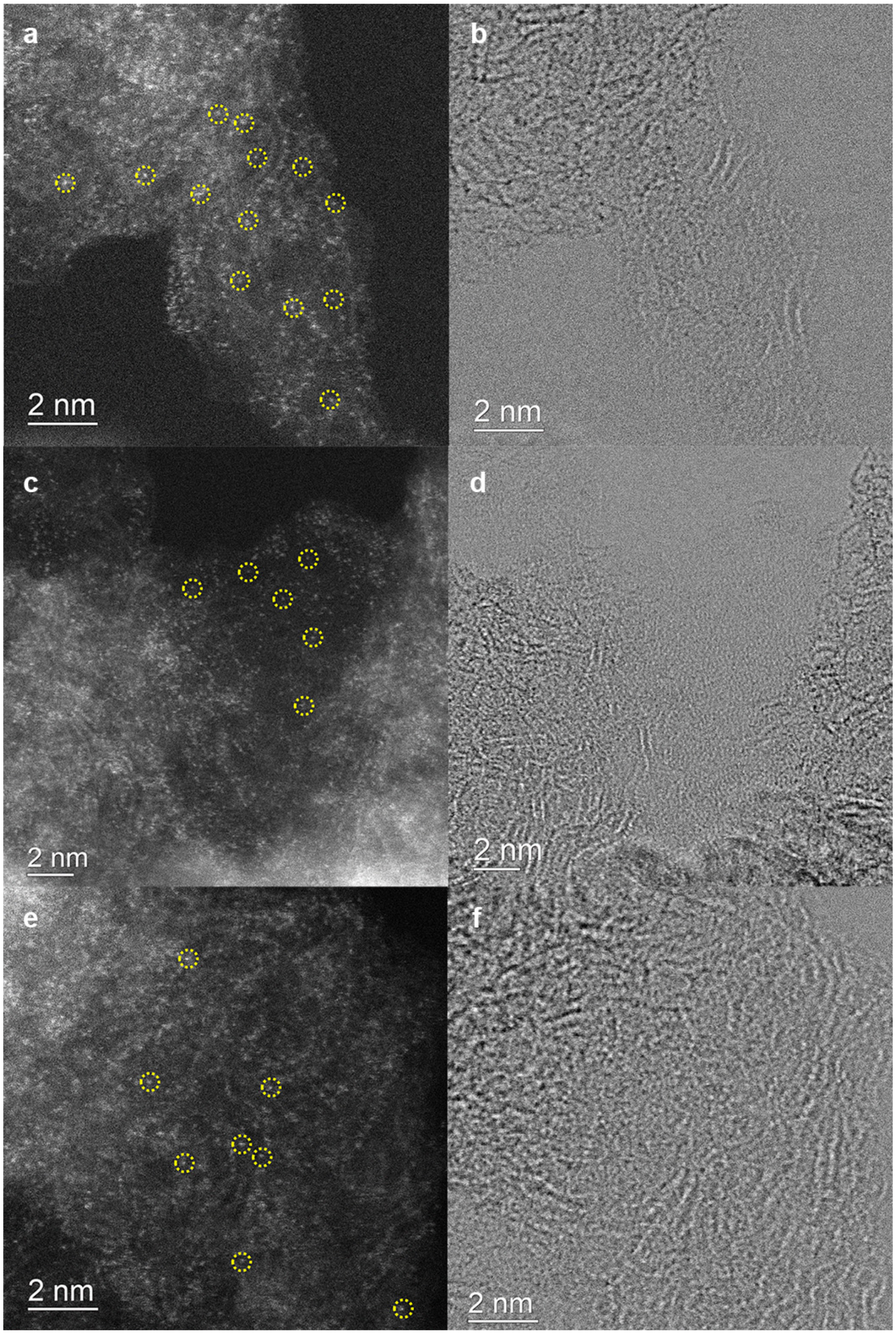

Supplementary Figure 7. Characterize the Fe single atoms in the Fe-N_{900}-PCC. (a, c, e) Representative AC-STEM images, Fe single atoms are highlighted by yellow circles. **(b, d, f)** Corresponding HRTEM images.

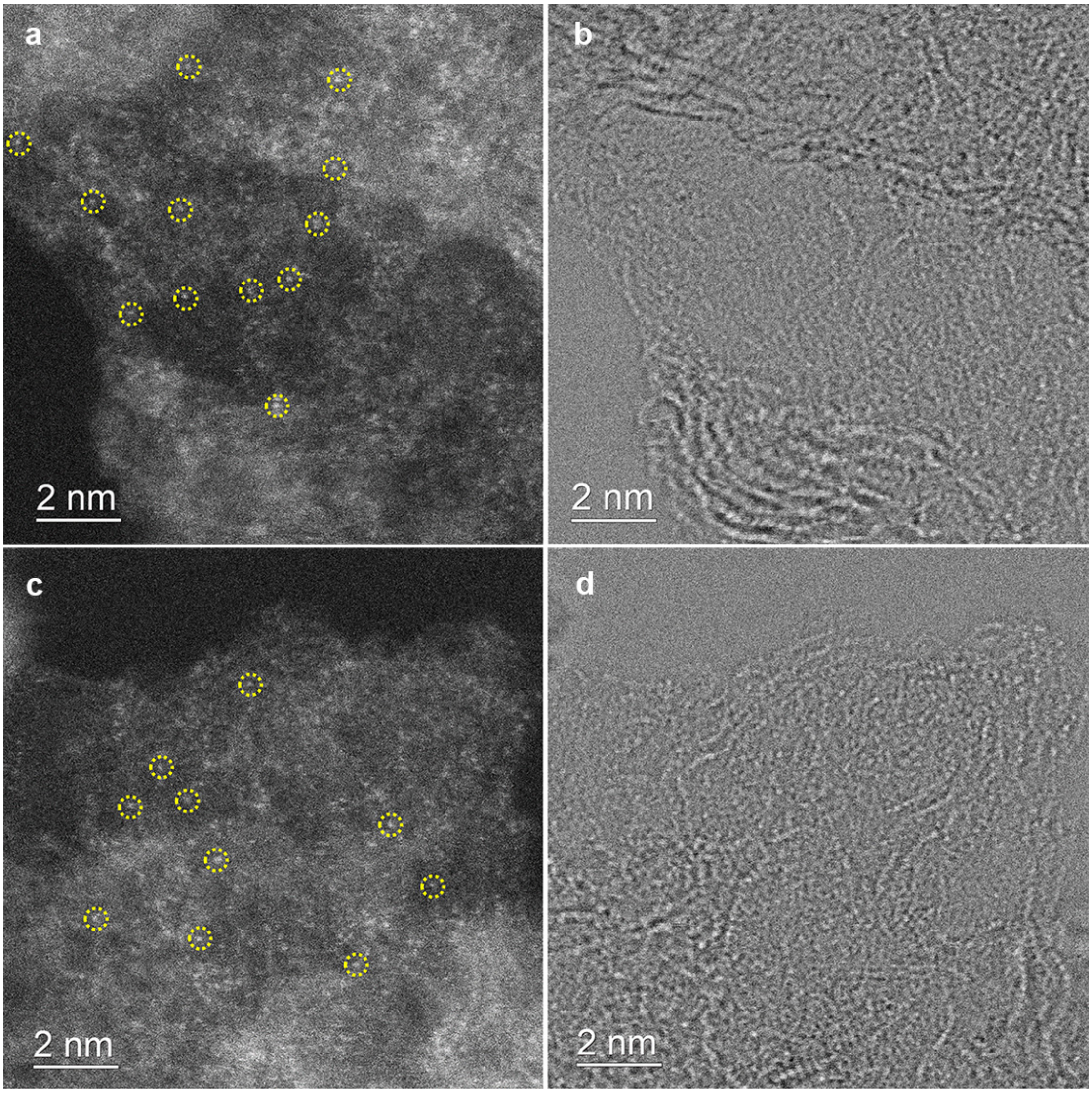

Supplementary Figure 8. Characterize the Fe single atoms in the Fe-P$_{900}$-PCC. (a, c) Representative AC-STEM images, Fe single atoms are highlighted by yellow circles. **(b, d)** Corresponding HRTEM images.

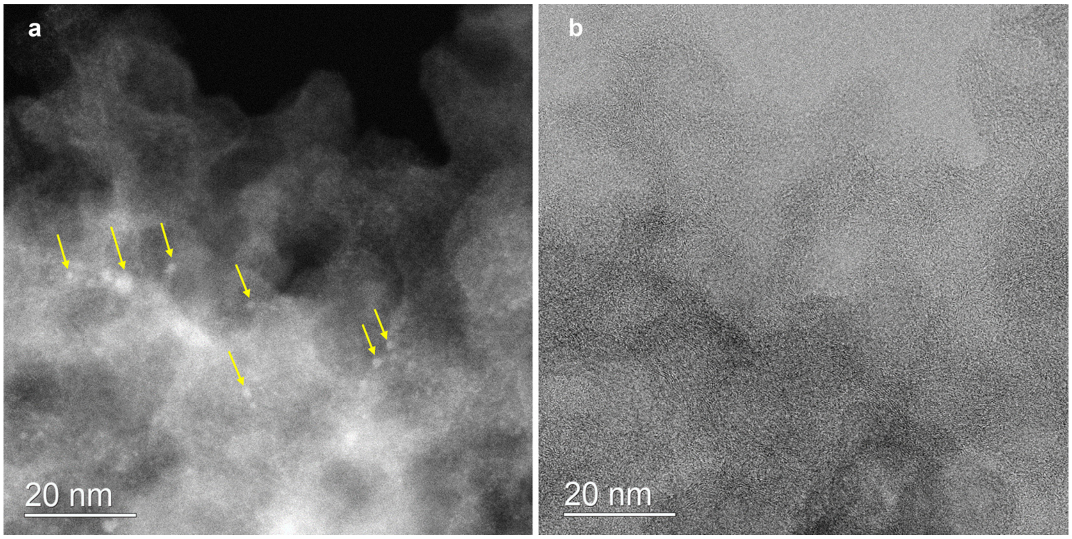

Supplementary Figure 9. Characterize the Fe nanoparticles in the Fe-N_{900}-PCC. (**a**) STEM image, Fe nanoparticles are highlighted by yellow arrows. (**b**) Corresponding HRTEM image.

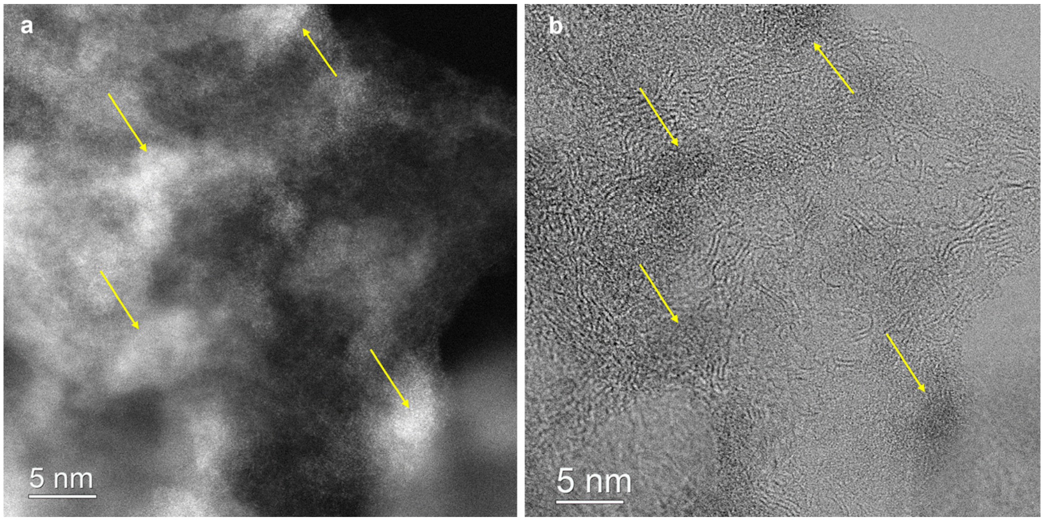

Supplementary Figure 10. Characterize the Fe nanoparticles in the Fe-P_{900}-PCC. (a) STEM image. **(b)** Corresponding HRTEM image. Fe nanoparticles are highlighted by yellow arrows.

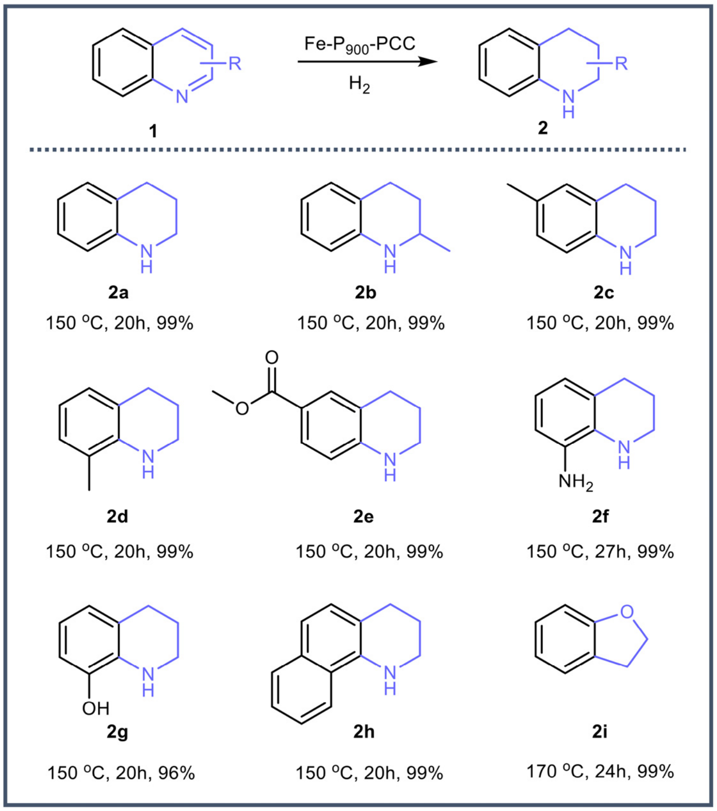

Supplementary Figure 11. Exploration of substrate scope for the hydrogenation of unsaturated N-heterocycles. Reaction conditions: 1 mmol substrate, 100 mg Fe-P_{900}-PCC, 2 mL solvent (heptane), 4 MPa H_2. Yields were determined by GC using dodecane as an internal standard.

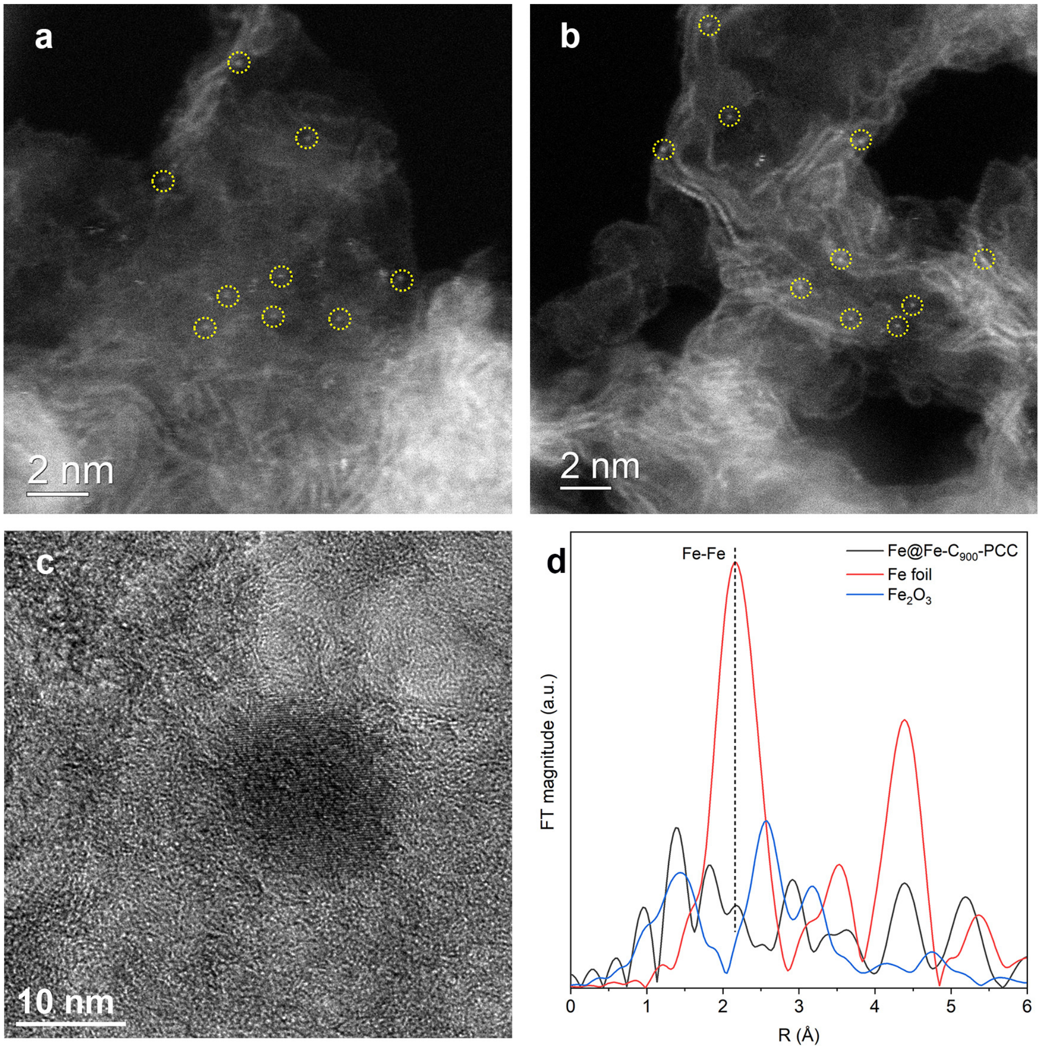

Supplementary Figure 12. Characterizations of the Fe@Fe-C_{900}-PCC. (**a**, **b**) AC-STEM images, Fe single atoms are highlighted by yellow circles. (**c**) HRTEM image. (**d**) Fe K-edge EXAFS spectra of Fe@Fe-C_{900}-PCC and reference materials (Fe foil and Fe_2O_3).

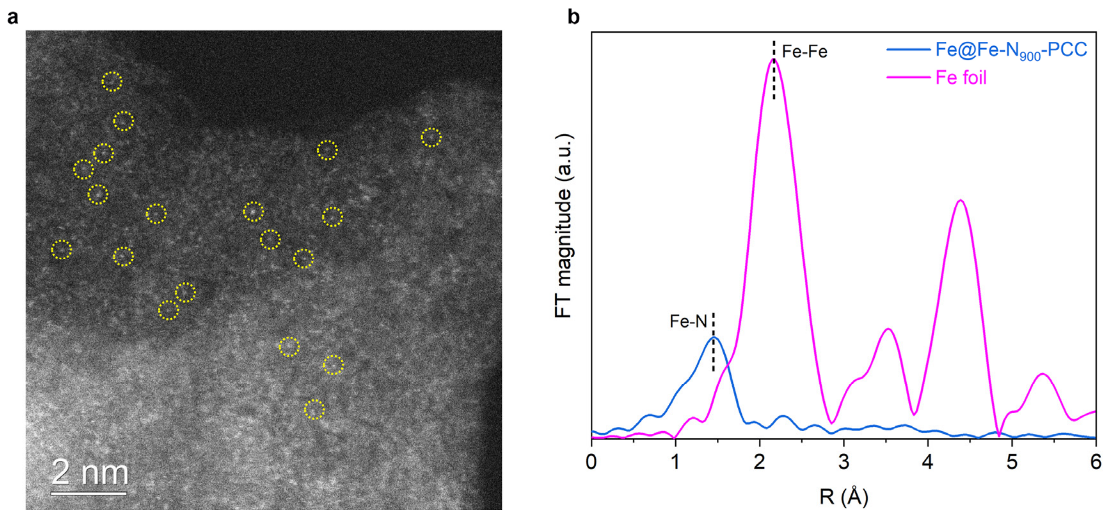

Supplementary Figure 13. Characterizations of the Fe@Fe-N$_{900}$-PCC. (**a**) AC-STEM image, Fe single atoms are highlighted by yellow circles. (**b**) Fe K-edge EXAFS spectra of Fe@Fe-N$_{900}$-PCC and Fe foil.

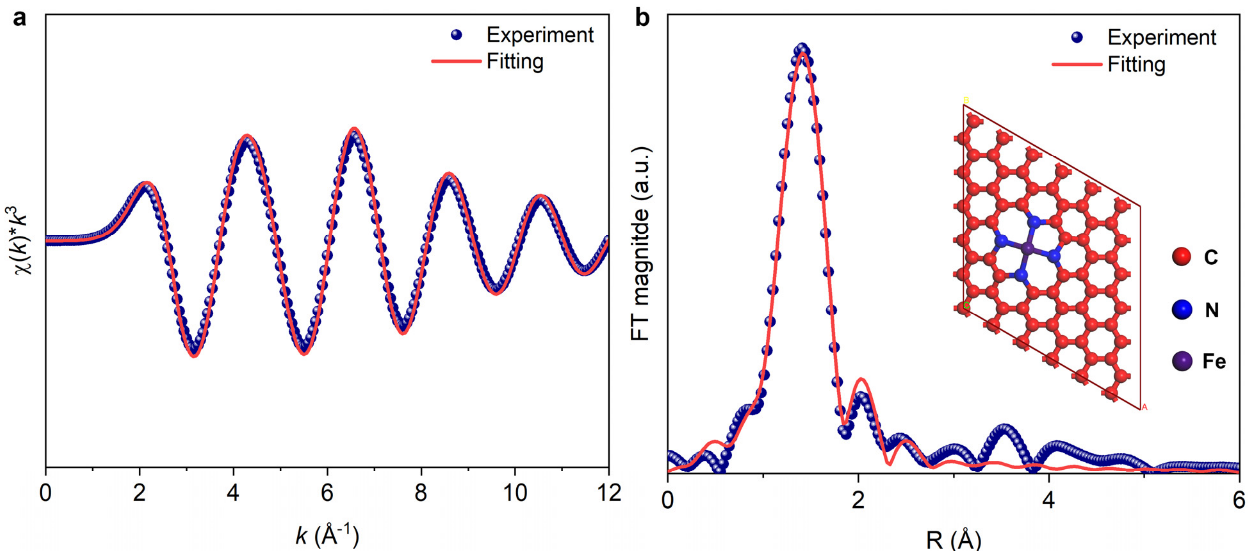

Supplementary Figure 14. Chemical environment of the Fe@Fe-N_{900}-PCC. Fe K-edge EXAFS analysis of the Fe@Fe-N_{900}-PCC at *k*-space (**a**) and R-space (**b**), respectively. The inset in (**b**) demonstrates the schematic model of Fe-N_4. The best-fit structural parameters are listed in Supplementary Table 6.

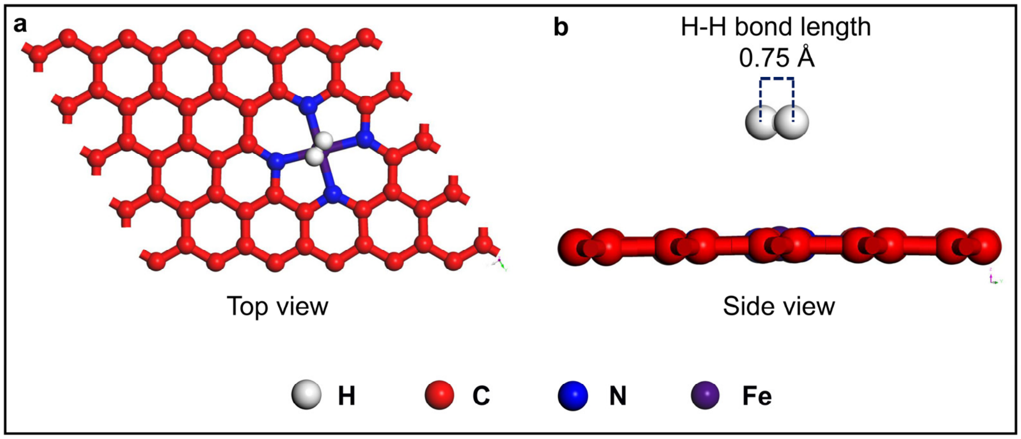

Supplementary Figure 15. Adsorption configurations of H_2 molecule on Fe-N_4 structure. (a) Top view. **(b)** Side view.

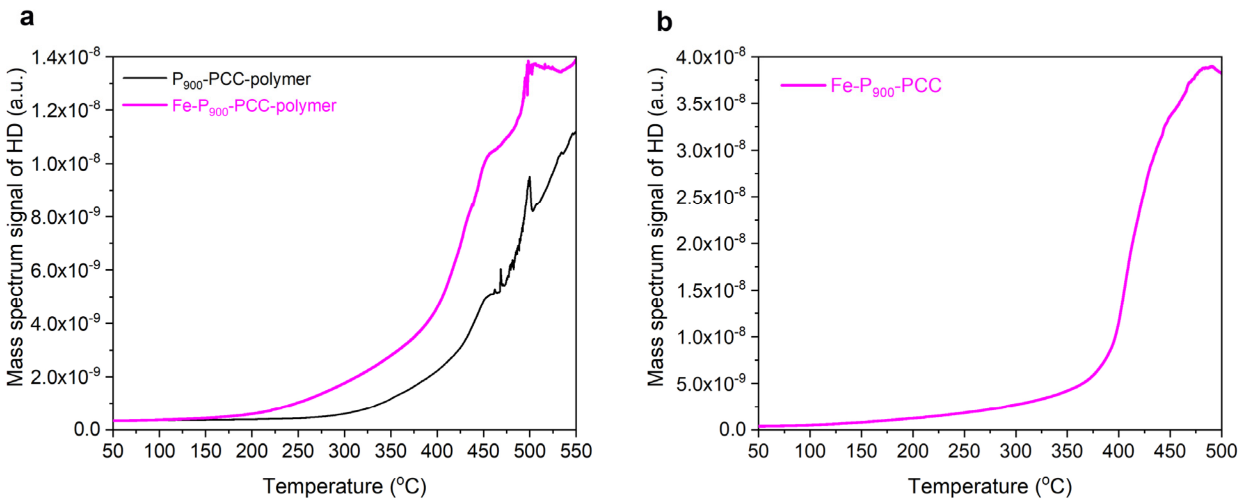

Supplementary Figure 16. Gas-phase isotopic H_2-D_2 exchange experiments. (**a**) HD profiles of polymer derived catalysts (P_{900}-PCC-polymer, Fe-P_{900}-PCC-polymer). (**b**) HD profile of Fe-P_{900}-PCC.

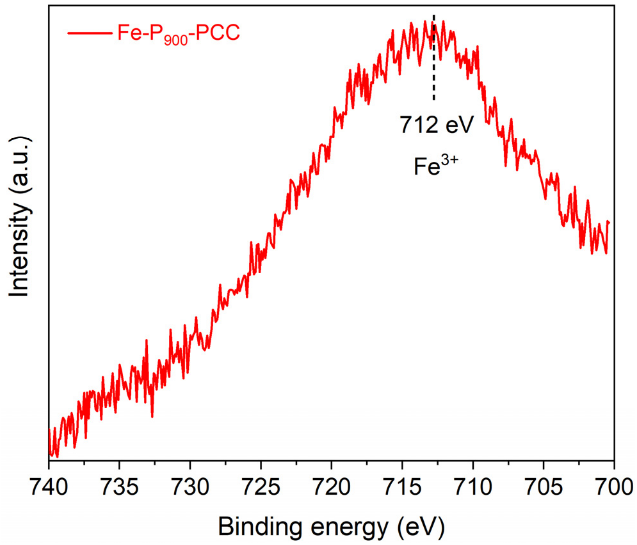

Supplementary Figure 17. Fe 2p XPS spectrum of the Fe-P_{900}-PCC. The black vertical line indicate the binding energy of Fe 2p3/2 of Fe^{3+}.

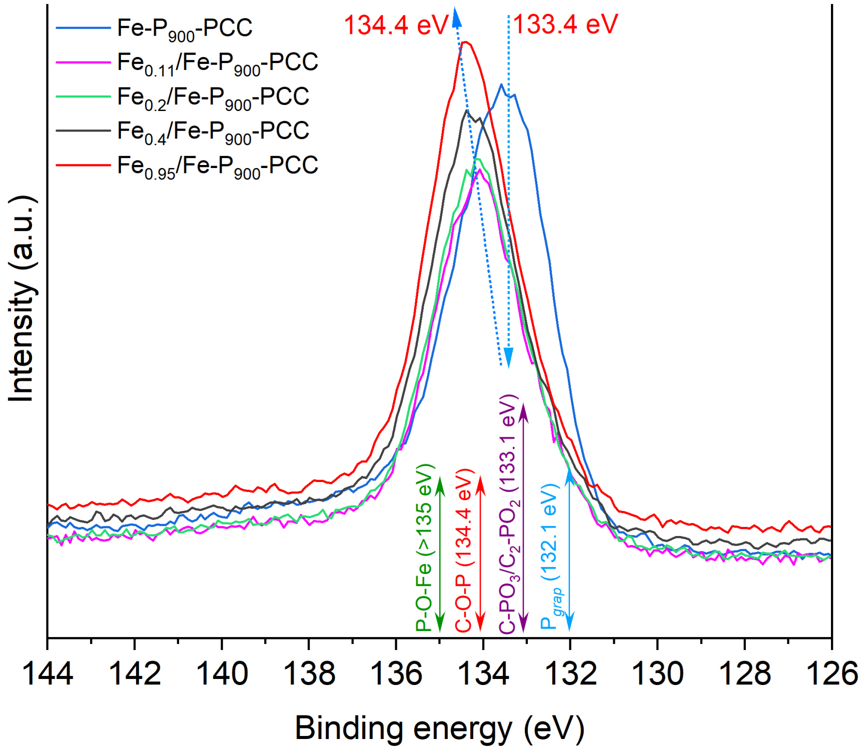

Supplementary Figure 18. P 2p XPS spectra of Fe-P$_{900}$-PCC and Fe$_x$/Fe-P$_{900}$-PCC. The P 2p XPS spectra of Fe-P$_{900}$-PCC have been changed by post-impregnation Fe species.

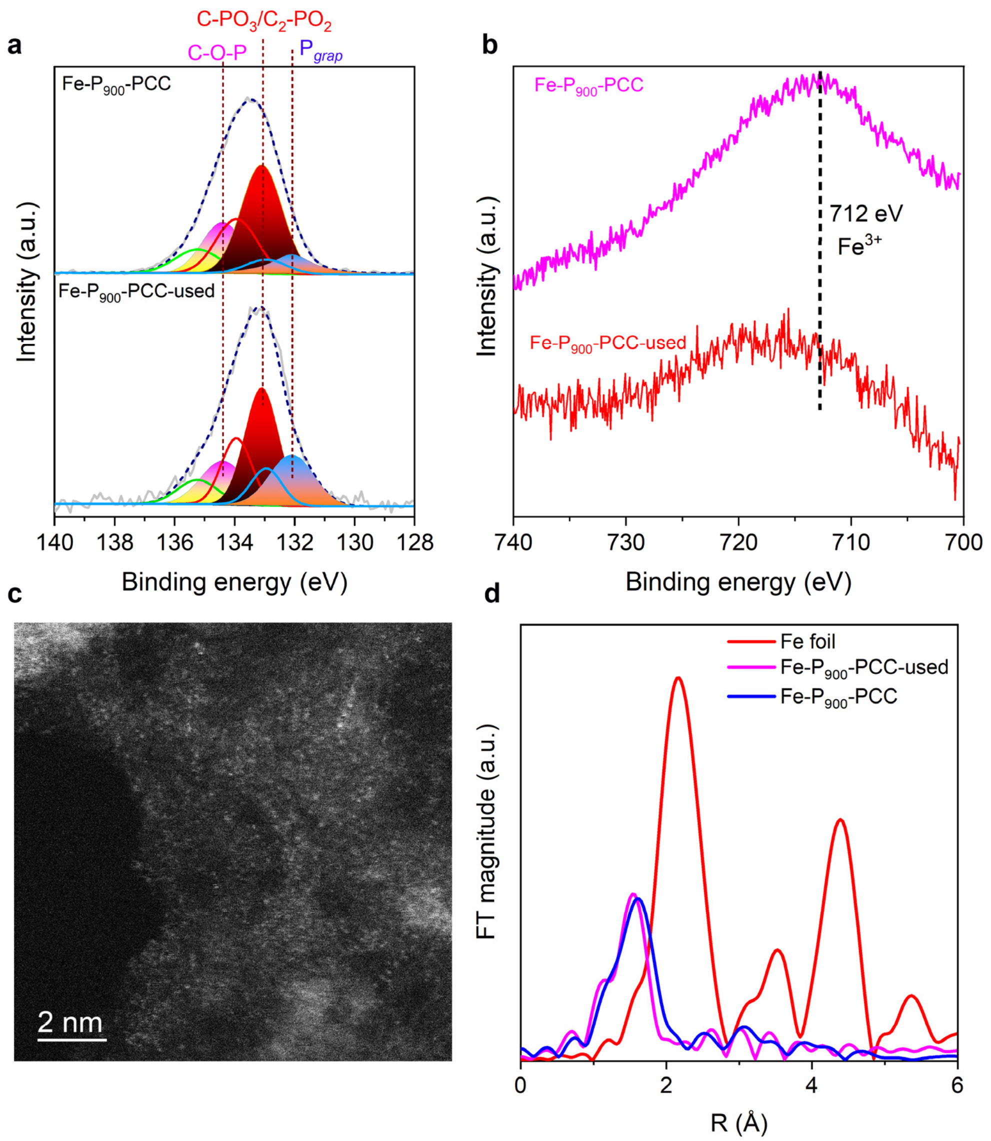

Supplementary Figure 19. Characterizations of spent Fe-P900-PCC. (a) P 2p XPS spectra, the contents of different P species are listed in Supplementary Table 5. **(b)** Fe 2p XPS spectra. **(c)** STEM image of Fe-P900-PCC-used. (**d**) Fe K-edge EXAFS spectra of Fe-P900-PCC and Fe-P900-PCC-used, as well as the reference sample Fe foil.

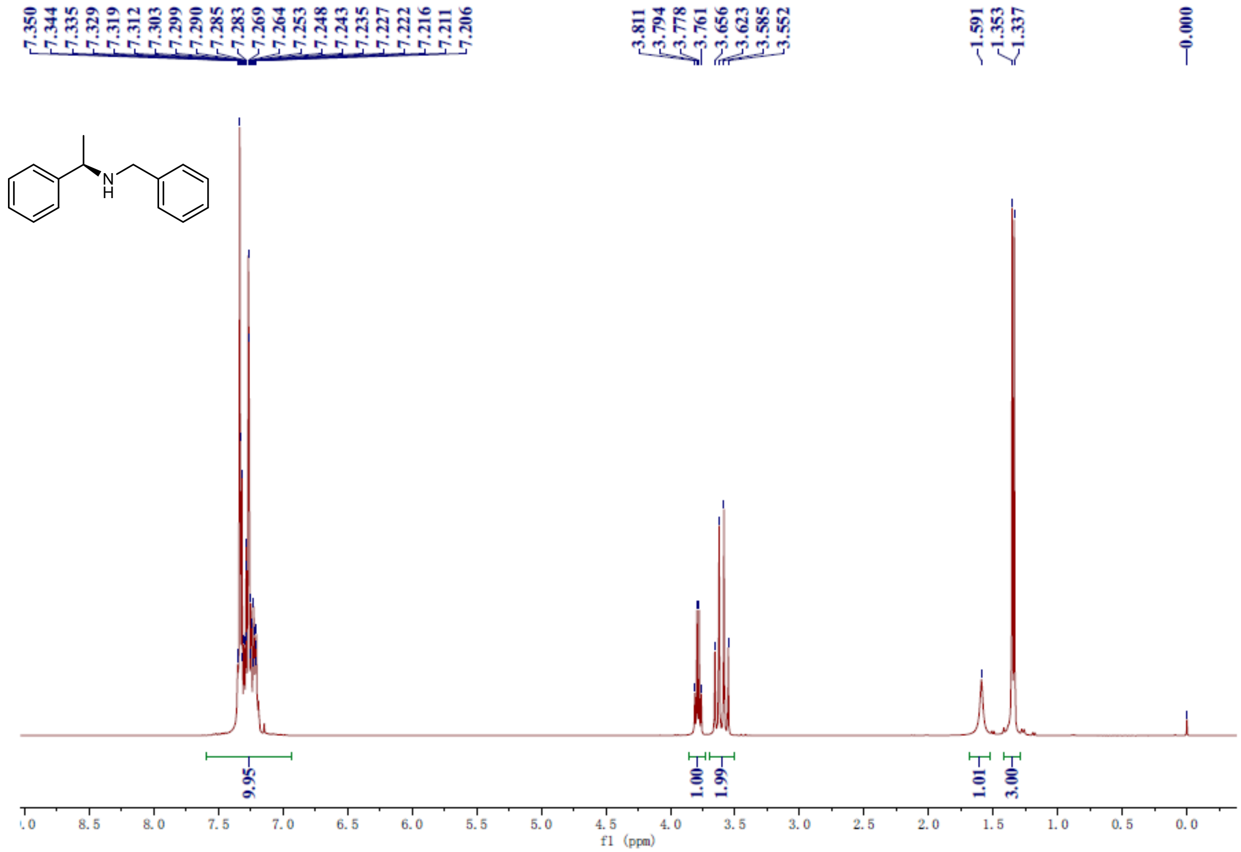

Supplementary Figure 20. ^{1}H NMR spectrum of compound **5n**.

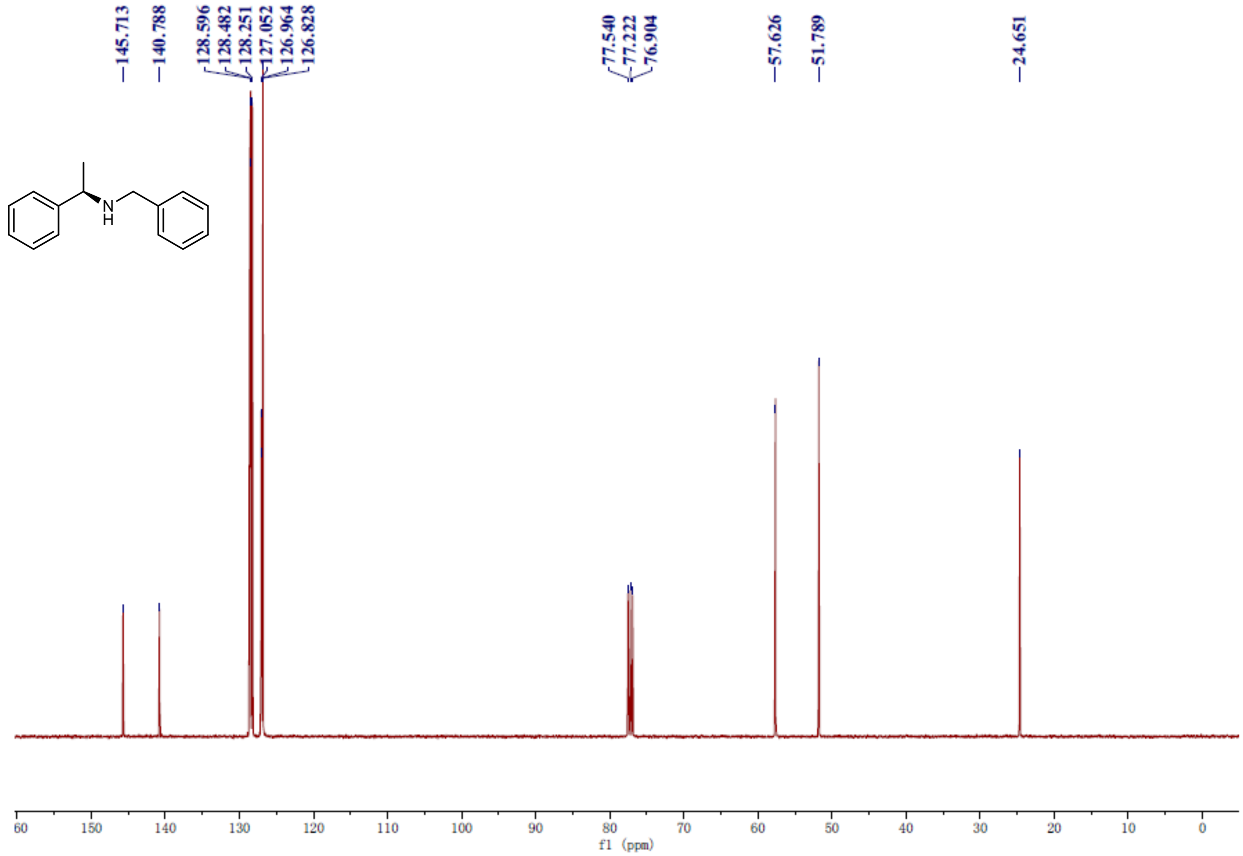

Supplementary Figure 21. ^{13}C NMR spectrum of compound **5n**.

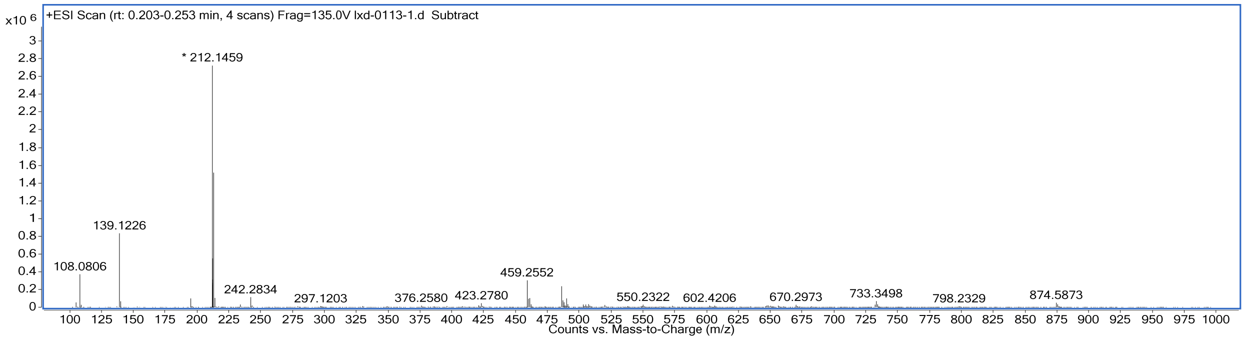

Supplementary Figure 22. HRMS of compound **5n**.

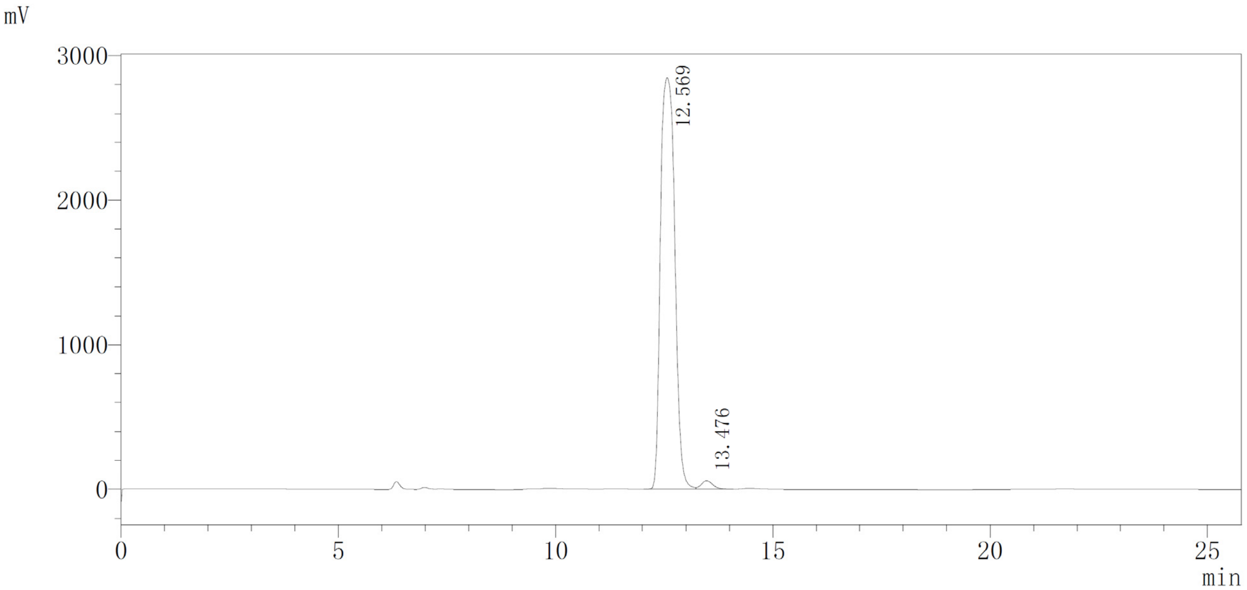

Supplementary Figure 23. Chiral HPLC data of compound **5n**. Peak 1: 12.596 min, area (65198511 mAU*s), area percentage (98.364%); peak 2: 13.476 min, area (1084165 mAU*s), area percentage (1.636%).

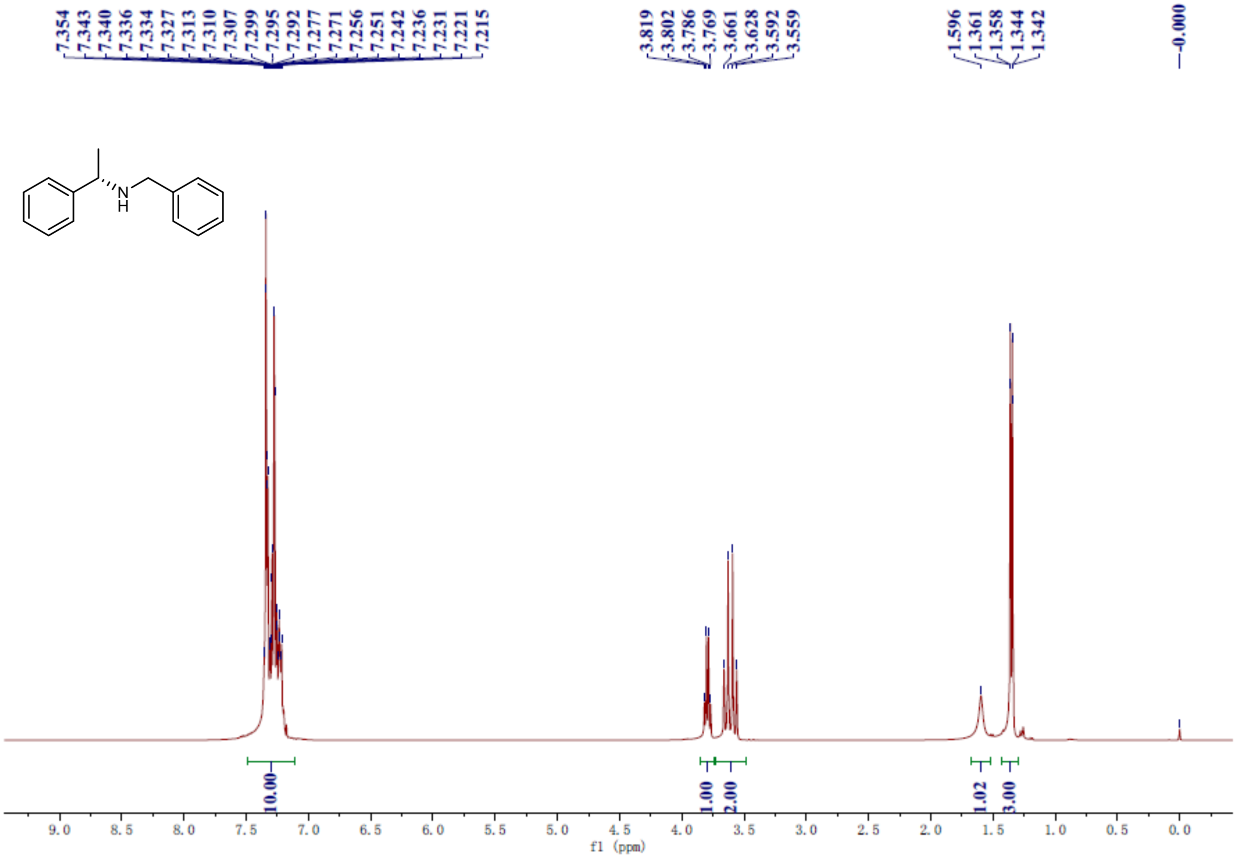

Supplementary Figure 24. ^{1}H NMR spectrum of compound **5o**.

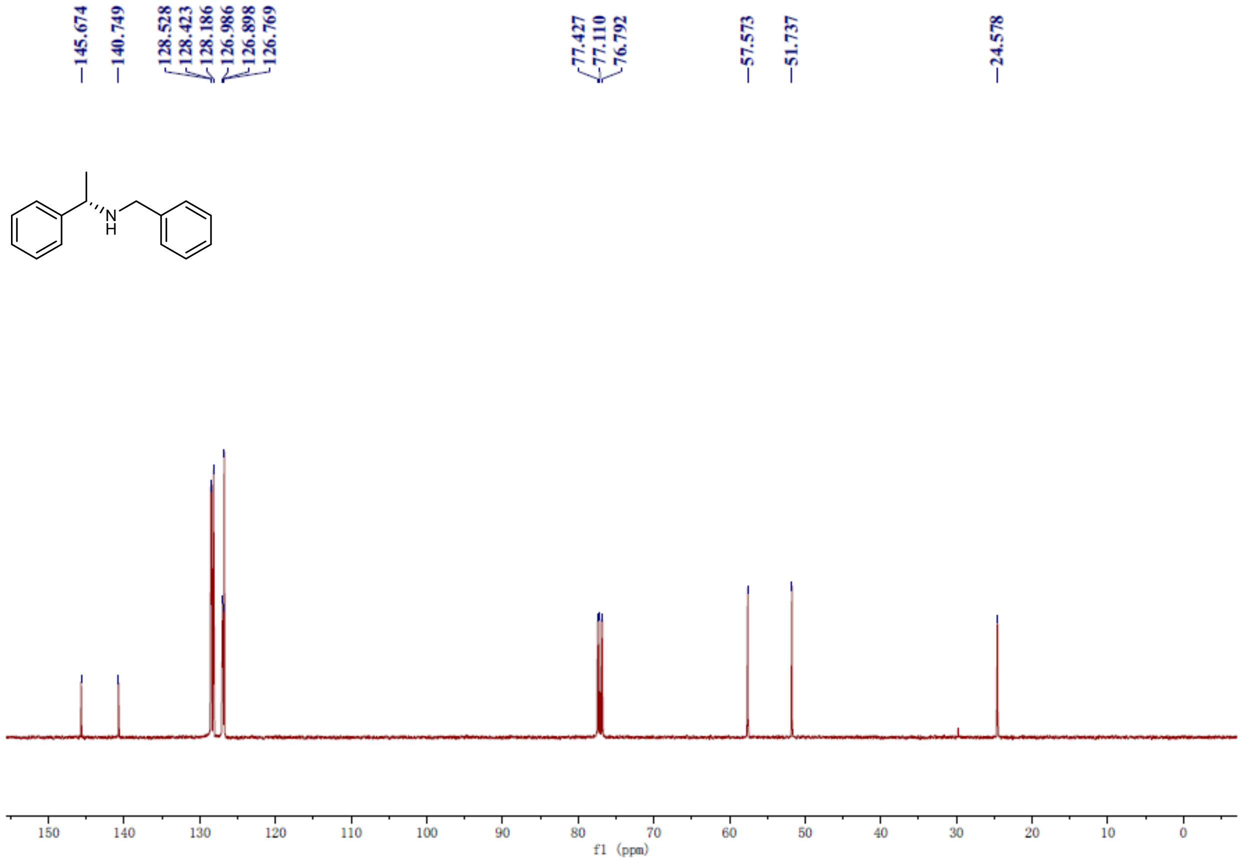

Supplementary Figure 25. ^{13}C NMR spectrum of compound **5o**.

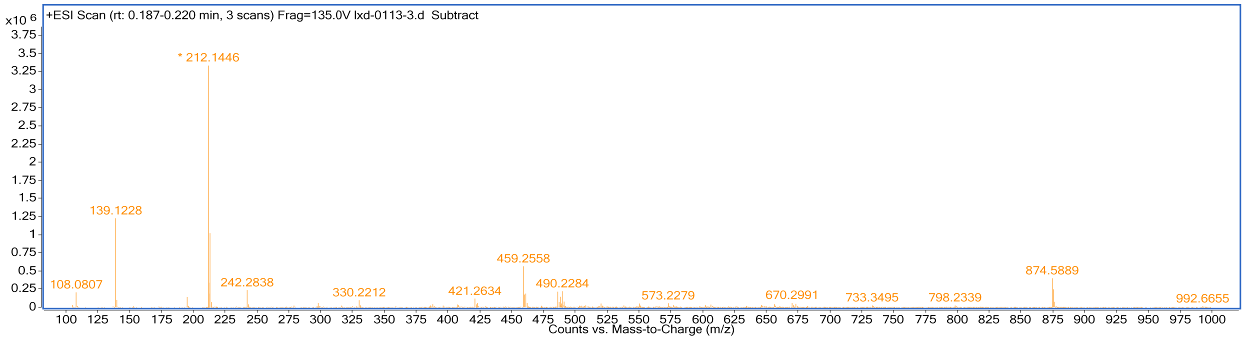

Supplementary Figure 26. HRMS of compound **5o**.

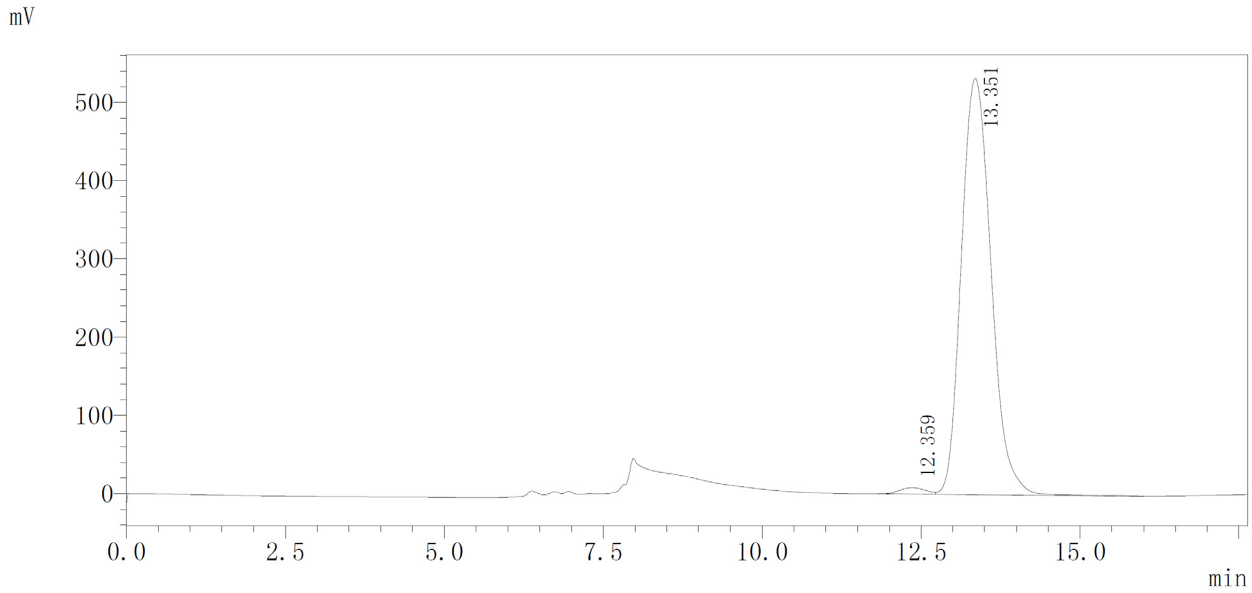

Supplementary Figure 27. Chiral HPLC data of compound **5o**. Peak 1: 12.359 min, area (234963 mAU*s), area percentage (1.339%); peak 2: 13.351 min, area (17318351 mAU*s), area percentage (98.661%).

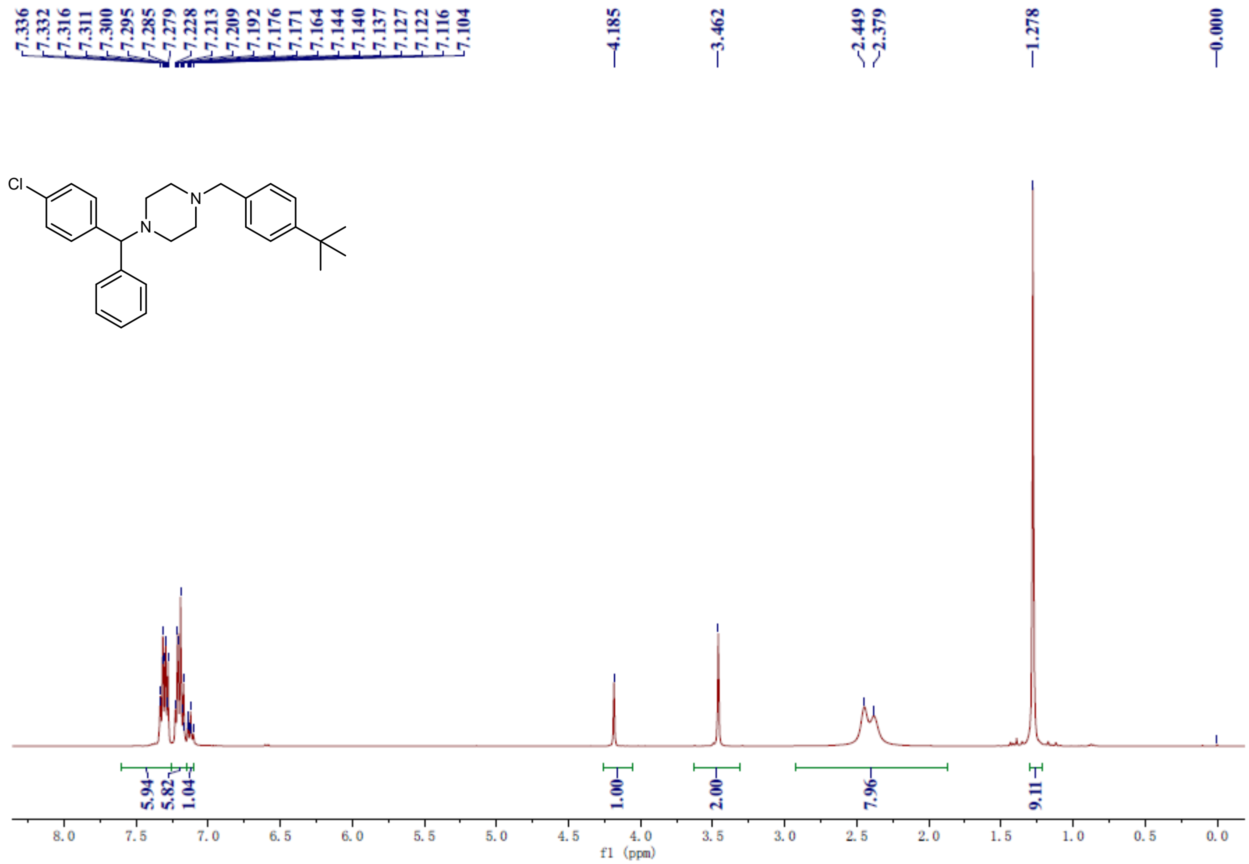

Supplementary Figure 28. ^{1}H NMR spectrum of compound **5p**.

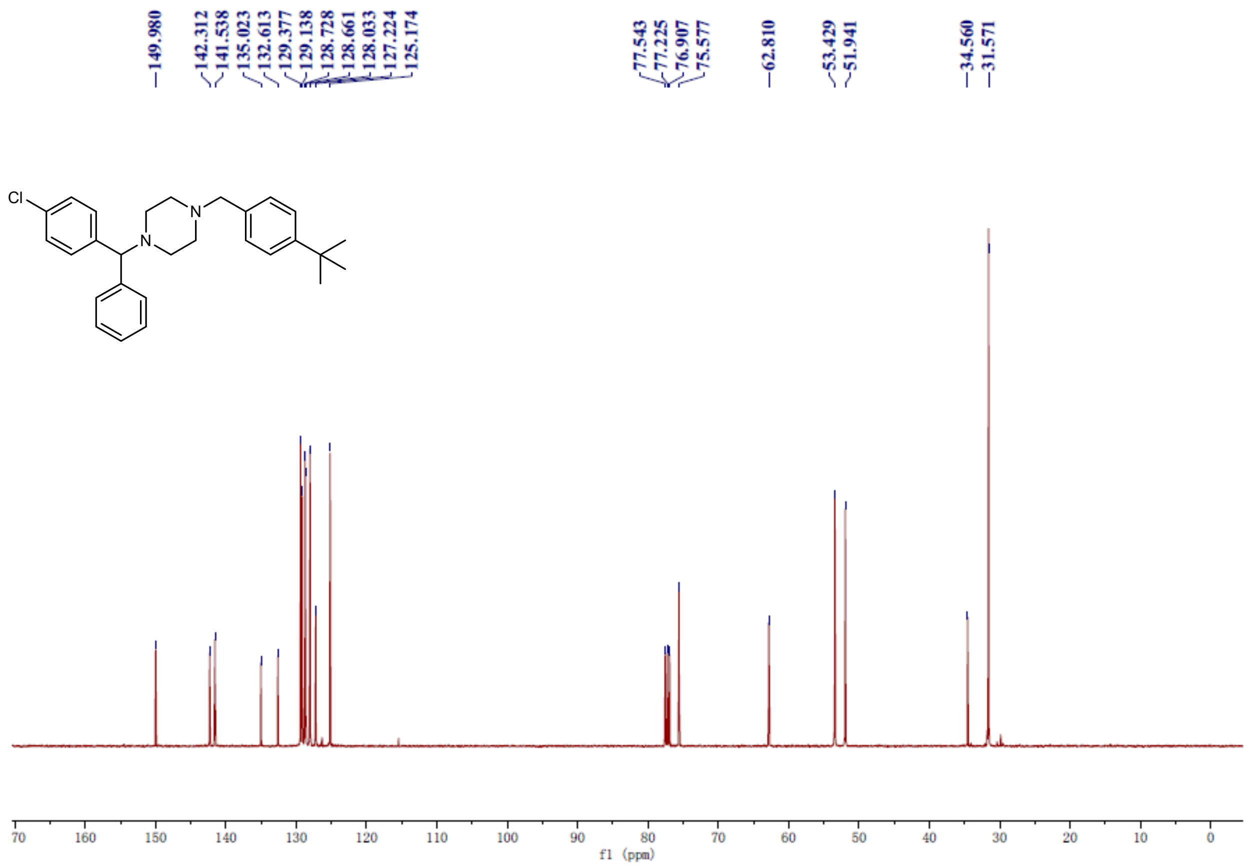

Supplementary Figure 29. ^{13}C NMR spectrum of compound **5p**.

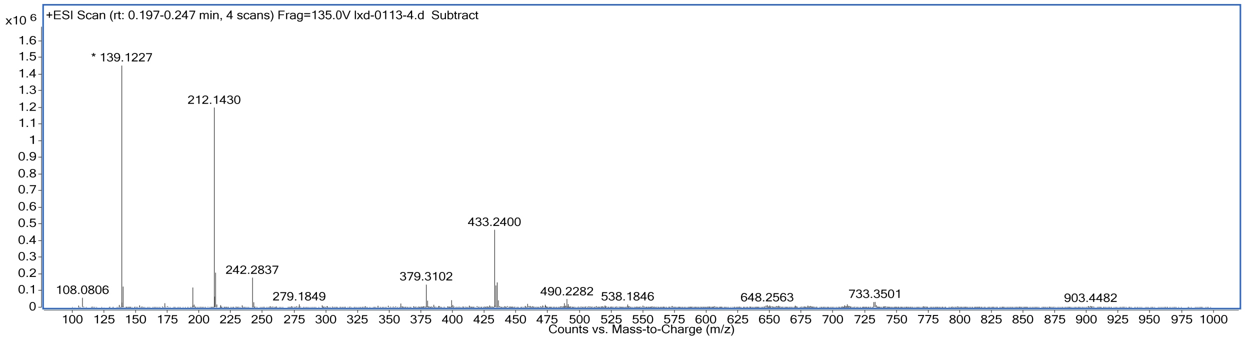

Supplementary Figure 30. HRMS of compound **5p**.

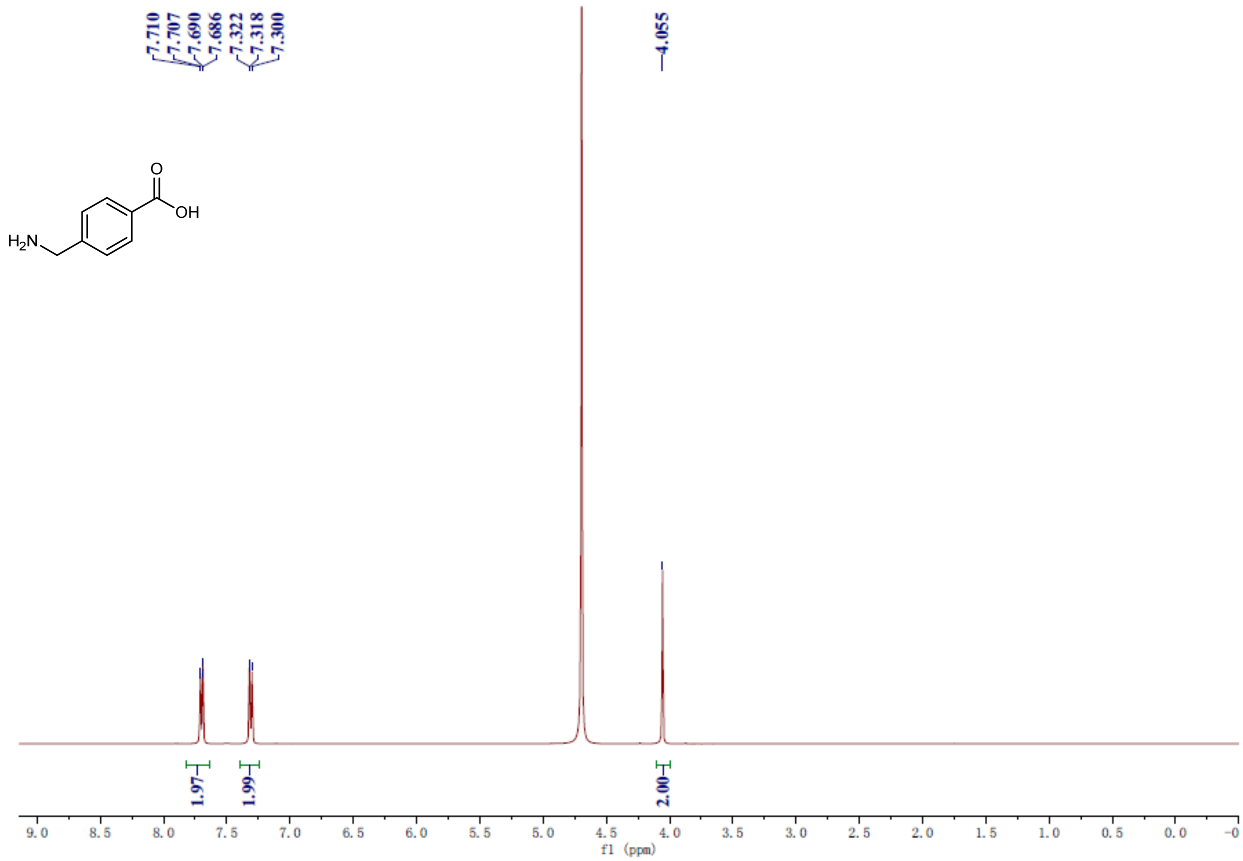

Supplementary Figure 31. ^{1}H NMR spectrum of compound **5q**.

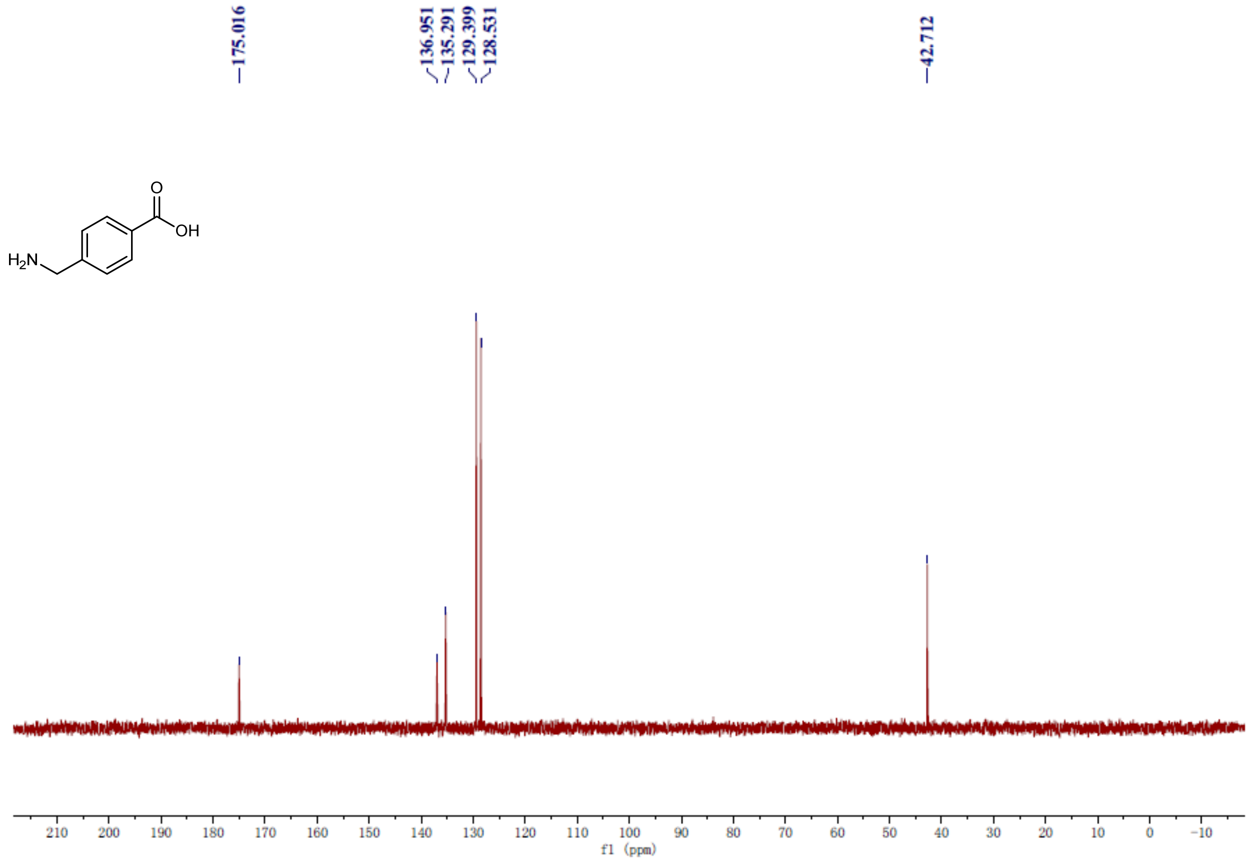

Supplementary Figure 32. ^{13}C NMR spectrum of compound **5q**.

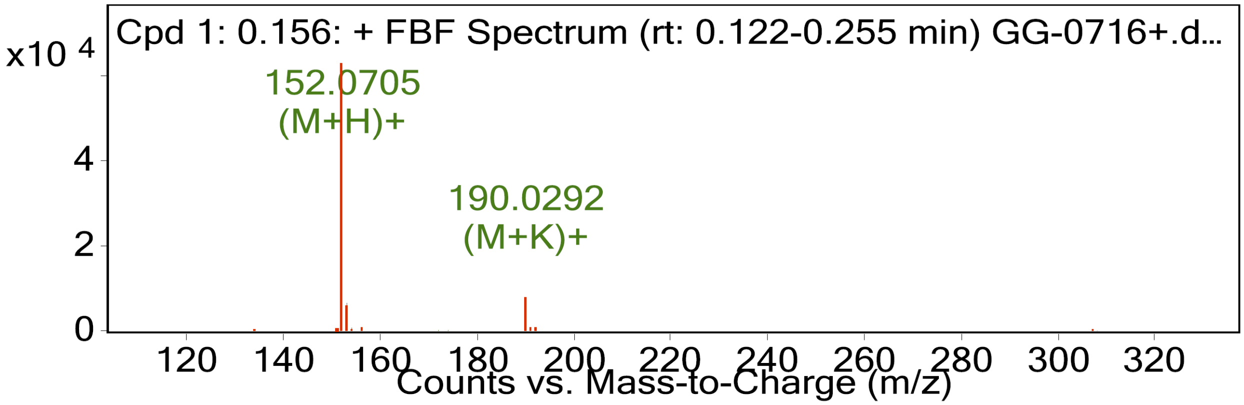

Supplementary Figure 33. HRMS of compound **5q**.

Supplementary Tables

Supplementary Table 1. The contents of Fe in raw materials.

Raw material	Fe content (wt%)
Silica colloid (Alfa Aesar)	3.5×10^{-4}
Sucrose (Macklin Biochemical Co. Ltd)	9.3×10^{-4}
Phytic acid solution (Aladdin Industrial Cooperation)	9.2×10^{-2}
Cyanamide (Alfa Aesar)	1.5×10^{-3}

Determined by ICP-MS. Company name of purchased raw materials are shown in parenthesis.

Supplementary Table 2. The contents of Fe in the catalysts.

Catalyst	Fe content (wt%)
Fe-C_{900}-PCC	1.6×10^{-3}
Fe-N_{900}-PCC	2.3×10^{-3}
Fe-P_{900}-PCC	7.1×10^{-2}
Fe@Fe-C_{900}-PCC	3.8×10^{-2}
Fe@Fe-N_{900}-PCC	5.7×10^{-2}
P_{900}-PCC-polymer	2.3×10^{-5}
Fe-P_{900}-PCC-polymer	9.6×10^{-2}
Fe-P_{700}-PCC	7.2×10^{-3}
Fe-P_{800}-PCC	3.5×10^{-2}
Fe-P_{1000}-PCC	1.1×10^{-1}
Fe-P_{1100}-PCC	6.8×10^{-2}

Determined by ICP-MS.

Supplementary Table 3. Elemental compositions of catalysts (XPS results).

Catalyst	XPS analysis (atomic %)					
	C	O	F	Fe	N	P
Fe-C_{900}-PCC	91.02	6.08	2.6	0.3	-	-
Fe-N_{900}-PCC	81.69	6.24	0.92	0.16	10.99	-
Fe-P_{700}-PCC	86.23	10.66	0.91	0.16	-	2.04
Fe-P_{800}-PCC	81.86	12.97	1.43	0.26	-	3.48
Fe-P_{900}-PCC	84.55	11.55	0.96	0.20	-	2.74
Fe-P_{1000}-PCC	86.81	9.68	1.2	0.26	-	2.04
Fe-P_{1100}-PCC	91.47	6.45	1.01	0.15	-	0.91
Fe-P_{900}-PCC-H	93.05	5.64	0.33	0.14	-	0.98

Supplementary Table 4. Structural properties of the catalysts.

Catalyst	S_{BET}† (m^2g^{-1})	S_{micro}‡ (m^2g^{-1})	V_{pore}# (m^3g^{-1})	V_{micro}§(m^3g^{-1})	D_{pore}¶ (nm)
Fe-C_{900}-PCC	768	263	1.34	0.14	12.7
Fe-N_{900}-PCC	628	193	1.11	0.10	12.7
Fe-P_{900}-PCC	511	259	1.32	0.14	17.2
Fe-P_{700}-PCC	480	243	1.13	0.13	17.2
Fe-P_{800}-PCC	496	251	1.29	0.13	17.2
Fe-P_{1000}-PCC	418	167	1.16	0.09	17.2
Fe-P_{1100}-PCC	615	275	1.45	0.14	17.2

†BET surface area.

‡Micropore surface area.

#Total pore volume.

§Pore volume for micropores.

¶Mean pore diameter.

Supplementary Table 5. The contents of different P species in catalysts.

Catalyst	Total P content (atomic %)	C-O-P (134.4 eV)		C-PO_3/C_2-PO_2 (133.1 eV)		P_{grap} (132.1 eV)	
		Percent-age (%)	Content (atomic %)	Percent-age (%)	Content (atomic %)	Percent-age (%)	Content (atomic %)
Fe-P_{700}-PCC	2.04	28.3	0.58	71.7	1.46	0	0
Fe-P_{800}-PCC	3.48	24.0	0.84	70.0	2.43	6.0	0.21
Fe-P_{900}-PCC	2.74	27.2	0.75	57.8	1.58	15.0	0.41
Fe-P_{1000}-PCC	2.04	31.2	0.64	51.4	1.05	17.4	0.35
Fe-P_{1100}-PCC	0.91	23.1	0.21	54.2	0.49	22.7	0.21
Fe-P_{900}-PCC-H	0.98	16.1	0.16	43.7	0.43	40.2	0.39
Fe-P_{900}-PCC-used	1.51	23.5	0.35	49.0	0.75	27.5	0.41

Content of $P_{species}$ (atomic %) = Total P content (atomic %) × Percentage of $P_{species}$ (%)

Supplementary Table 6. Fitting results of Fe K-edge EXAFS data for Fe@Fe-N_{900}-PCC and Fe-P_{900}-PCC.

Sample	Bond	CN	R (Å)	σ^2 ($Å^2$)	ΔE_0 (eV)	R-factor
Fe@Fe-N_{900}-PCC†	Fe-N	4.2 ± 0.4	1.95 ± 0.01	0.007 ± 0.001	-6.23 ± 1.1	0.004
Fe-P_{900}-PCC‡	Fe-P	4.0 ± 0.8	2.35 ± 0.02	0.014 ± 0.006	-0.96 ± 0.3	0.013
	Fe-O	2.0 ± 0.4	2.00 ± 0.03	0.004 ± 0.004	-0.96 ± 0.3	

The average lengths of Fe-N, Fe-P and Fe-O bonds and coordination numbers of Fe atoms are extracted from the curve fitting for Fe K-edge EXAFS data. CN, coordination number; R, distance between absorber and backscatter atoms; σ^2, the Debye-Waller factor; ΔE_0, inner potential correction; R-factor, indicate the goodness of the fit.

†For the EXAFS spectrum of Fe@Fe-N_{900}-PCC (Supplementary Fig. 14), only a strong Fe-N peak at 1.45 Å is observed. So, the fitting was performed by including a single Fe-N shell within the R-rang of 1.0 - 3.1 Å and *k*-rang of 1.42 $Å^{-1}$ - 9.62 $Å^{-1}$. The fitting results reveal that the coordination number of Fe center with surrounding N atoms is 4.2 ± 0.4 and the average Fe-N bond length is 1.95 ± 0.01 Å, suggesting the single Fe sites in Fe@Fe-N_{900}-PCC adopt a planar Fe-N_4 structure (as presented in Supplementary Fig. 14b).

‡The EXAFS spectrum of Fe-P_{900}-PCC shows that the main peak locates at 1.63 Å, ascribing to Fe-P first shell coordination. Furthermore, the Fe-O first shell coordination at 1.45 Å is also included in this broadening peak, which indicates that O need to be included in the curve fitting. On the other hand, a shoulder peak at 2.55 Å for Fe-C second shell coordination is also observed. Therefore, a three-shell structure model, including a Fe-P, a Fe-O and a Fe-C shell, is initially used to fit the EXAFS data of Fe-P_{900}-PCC within the R-rang of 1.0 - 3.1 Å and *k*-rang of 1.42 - 9.62 $Å^{-1}$. The best-fitting analyses manifests that the dominant contribution is given by Fe-P and Fe-O first shell coordination as presented in Manuscript Fig. 3c and 3d. The coordination numbers for P and O atoms are calculated as 4.0 ± 0.8 and 2.0 ± 0.4, and the corresponding mean bond length of Fe-P and Fe-O are 2.35 ± 0.02 Å and 2.00 ± 0.03 Å, respectively. These results reveal that the single Fe atom in Fe-P_{900}-PCC coordinates with four P atoms and a dioxygen molecule (O_2-Fe-P_4). Because the atomic size of P (106 pm) is larger than C (75 pm), Fe center adopts a pyramidal geometry as shown in Manuscript Fig. 3e, this configuration is quite different from the planar structure of Fe-N_4.

Supplementary Table 7. Hydrogenation of quinoline catalyzed by Fe_x/Fe-P_{900}-PCC.

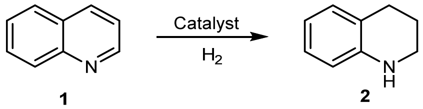

1 2

Entry	Catalyst	Temperature (ºC)	Conversion (%)	Yield (%)
1	$Fe_{0.11}$/Fe-P_{900}-PCC	150	19	18
2	$Fe_{0.2}$/Fe-P_{900}-PCC	150	13	11
3	$Fe_{0.4}$/Fe-P_{900}-PCC	150	7	7
4	$Fe_{0.95}$/Fe-P_{900}-PCC	150	5	5

Reaction conditions: 1 mmol quinoline, 100 mg catalyst, 2 mL solvent (heptane), 4 MPa H_2, 12 h. The conversion and yield were determined by GC using dodecane as an internal standard.

Supplementary Table 8. Step by step barrier (E_a, eV) and reaction energy (E_r, eV) for hydrogenation of quinoline (C_9H_7N) over Fe-P_{900}-PCC.

Number	Reactions	E_a (eV)	E_r (eV)
1	$H_2(g) \rightarrow H_2*$	-	-0.407
2	$C_9H_7N + H_2* \rightarrow C_9H_7N* + H_2*$	-	-0.687
3	$C_9H_7N* + H_2* \rightarrow C_9H_8N* + H(Fe)*$	0.220	-0.004
4	$C_9H_8N* + H(Fe)* \rightarrow C_9H_9N*$	0.380	-0.348
5	$C_9H_9N* + H_2(g) \rightarrow C_9H_9N* + H_2*$	-	-0.025
6	$C_9H_9N* + H_2* \rightarrow C_9H_{10}N* + H(Fe)*$	0.728	0.432
7	$C_9H_{10}N* + H(Fe)* \rightarrow C_9H_{11}N$	0.132	-1.331

Supplementary Table 9. The energies of species in the processes of hydrogenation of quinoline (C_9H_7N).

Label	Species	E (eV)	E_{rel} (eV)†
IS	$C_9H_7N + H_2(g)$	-635.885	0.000
int-1	$C_9H_7N + H_2*$	-643.051	-0.407
int-2	$C_9H_7N* + H_2*$	-758.800	-1.094
TS1	-	-	-
int-3	C_9H_8N* + H(Fe)*	-758.804	-1.098
TS2	-	-	-
int-4	C_9H_9N*	-759.152	-1.446
int-5	$C_9H_9N* + H_2*$	-765.936	-1.471
TS3	-	-	-
int-6	$C_9H_{10}N*$ + H(Fe)*	-765.504	-1.039
TS4	-	-	-
int-7	$C_9H_{11}N$	-766.836	-2.370
FS	-	-635.885	-1.801

†The E_{rel} refers to the energy of species labelled IS.

IS: initial state. int: intermediate. TS: transition state. FS: final state.

Supplementary Table 10. Catalytic performances for non-precious metal catalyzed heterogeneous hydrogenation of quinoline in earlier literatures.

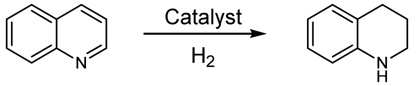

Entry	Catalyst	NP†/ SA‡	Reaction conditions	Yield (%)	TOF# (h^{-1})	Ref.
1	Fe-P_{900}-PCC	SA	150 °C, heptane, 4 MPa H_2, 12 h	92	60.4	This work
2	Co_3O_4-Co/NGr@α-Al_2O_3	NP	120 °C, toluene, 2 MPa H_2, 48 h	98	0.5	Ref[1]
3	Co_1/h-NC	SA	120 °C, THF, 3.5 MPa H_2, 10 h	56	5.6	Ref[2]
4	Co@NGS-800-NL	NP	140 °C, isopropanol, 4 MPa H_2, 24 h	96	0.4	Ref[3]
5	CoO_x@CN	NP	120 °C, methanol, 3.5 MPa H_2, 3 h	91	6.6	Ref[4]
6	Fe(1)/L4(4.5)@C-800(12)	NP	130 °C, isopropanol-H_2O, 4 MPa H_2, 56 h	87	0.1	Ref[5]
7	Ni NPs/[BMIM][Pro]	NP	75 °C, ethanol, 3 MPa H_2, 10 h	99	28.8	Ref[6]

†Nanoparticle catalyst
‡Single atom catalyst
#TOF = $mol_{\text{yield of tetrahydroquinoline}}$ / (mol_{metal} • h)

Supplementary Table 11. Catalytic performances for non-precious metal catalyzed heterogeneous hydrogenation of nitrobenzene in earlier literatures.

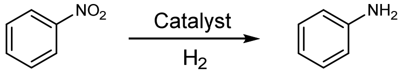

Entry	Catalyst	NP†/SA‡	Reaction conditions	Yield (%)	TOF# (h^{-1})	Ref.
1	Fe-P_{900}-PCC	SA	100 ºC, toluene, 4 MPa H_2, 18 h	99	43.7	This work
2	Fe-phen/C-800	NP	120 ºC, H_2O-THF, 5 MPa H_2,15 h	98	1.5	Ref[7]
3	Co-L1/carbon	NP	110 ºC, H_2O, 5 MPa H_2, 4 h	99	24.8	Ref[8]
4	Co@mesoNC	SA	110 ºC, ethanol, 3 MPa H_2, 2 h	55	42	Ref[9]
5	Co-SiCN	NP	110 ºC, ethanol-H_2O, 5 MPa H_2, 15 h	99	1.4	Ref[10]
6	CoO_x@NCNTs	NP	110 ºC, ethanol, 3 MPa H_2, 3 h	99	8.3	Ref[11]
7	Co_3O_4/NGr@C	NP	110 ºC, THF-H_2O, 5 MPa H_2, 4 h	95	25	Ref[12]
8	Fe-N-C@CNTs-1.5	NP	110 ºC, THF-H_2O, 5 MPa H_2, 6 h	99	46.8	Ref[13]
9	Fe_3C@G-CNT-700	NP	40 ºC, ethanol, 2 MPa H_2, 4.5 h	98	22	Ref[14]
10	Fe/N-C-500	NP	120 ºC, ethyl acetate, 4 MPa H_2, 15 h	99	0.6	Ref[15]
11	Co-Co_3O_4@carbon-700	NP	110 ºC, ethanol-H_2O, 4 MPa H_2, 15 h	99	3.9	Ref[16]
12	Fe_2O_3@G-C-900	NP	70 ºC, ethanol, 2 MPa H_2, 2 h	95	46.6	Ref[17]
13	Co@NC-800	NP	110 ºC, ethanol, 3 MPa H_2, 3 h	99	8	Ref[18]
14	Co@NMC-800	NP	80 ºC, ethanol, 1 MPa H_2, 80 min	99	37.5	Ref[19]
15	Co_2P/CN_x	NP	60 ºC, THF-H_2O, 5 MPa H_2, 6 h	99	1.5	Ref[20]
16	Zr_{12}-TPDC-CoCl	SA	110 ºC, toluene, 4 MPa H_2, 42 h	99	4.8	Ref[21]
17	Ni/SiO_2	NP	110 ºC, ethanol, 2.5 MPa H_2, 7 h	99	1.2	Ref[22]
18	Ni@PS_{60}SiCN	NP	110 ºC, ethanol-H_2O, 5 MPa H_2, 20 h	99	5	Ref[23]
19	7.2%Ni/Mo_2C	NP	80 ºC, ethanol-H_2O, 2 MPa H_2, 1.5 h	99	32.3	Ref[24]
20	Ni/C-300	NP	140 ºC, ethanol, 2 MPa H_2, 2 h	71	17.7	Ref[25]
21	Ni/AC_{OX}	NP	40 ºC, toluene, 0.3 MPa H_2, 190 min	95	1.8	Ref[26]
22	30.0 wt% Ni/C_{60}-Ac-B-4	NP	110 ºC, ethanol, 2 MPa H_2, 5 h	99	6.3	Ref[27]
23	Ni-NiO/NGr@C	NP	110 ºC, THF-H_2O, 5 MPa H_2, 8 h	98	2.5	Ref[28]
24	Ni/NGr@OMC-800	NP	100 ºC, H_2O, 5 MPa H_2, 2 h	99	17.2	Ref[29]
25	Ni-phen@SiO_2-1000	NP	40 ºC, methanol-H_2O, 1 MPa H_2, 20 h	99	1.3	Ref[30]

†Nanoparticle catalyst
‡Single atom catalyst
#TOF = $mol_{yield\ of\ aniline}$ / (mol_{metal} • h)

Supplementary Table 12. Catalytic performances for non-precious metal catalyzed heterogeneous reductive amination of carbonyl compounds in earlier literatures.

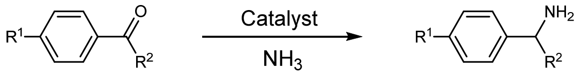

Entry	Catalyst	NP[†]/SA[‡]	Reaction conditions	Yield (%)	TOF[#] (h^{-1})	Ref.
1[a]	Fe-P_{900}-PCC	SA	75 °C, H_2O, 6 MPa H_2, 30 h	98	173	This work
2[b]	Co-DABCO-TPA@C-800	NP	120 °C, t-BuOH, 4 MPa H_2, 15 h	88	1.7	Ref[31]
3[c]	Ni-TA@SiO_2-800	NP	120 °C, t-BuOH, 2 MPa H_2, 24 h	98	0.7	Ref[32]
4[d]	Ni/gama-Al_2O_3	NP	80 °C, H_2O, 1 MPa H_2, 20 h	99	4.2	Ref[33]
5[e]	Fe/(N)SiC	NP	130 °C, H_2O, 6.5 MPa H_2, 20 h	89	0.4	Ref[34]
6[f]	Fe/(N)SiC	NP	140 °C, H_2O, 6.5 MPa H_2, 20 h	99	0.5	Ref[34]
7[g]	Co/N-C-800	NP	110 °C, H_2O, 0.5 MPa H_2, 4 h	92	1.8	Ref[35]
8[h]	Raney Ni	-	120 °C, methanol, 1 MPa H_2, 2 h	65	1.0	Ref[36]
9[i]	Raney Co	-	120 °C, methanol, 1 MPa H_2, 2 h	98	3.1	Ref[36]
10[j]	Ni_6AlO_x	NP	100 °C, H_2O, 0.1 MPa H_2, 6 h	99	0.3	Ref[37]
11[k]	Co@NC-800	NP	130 °C, ethanol, 1 MPa H_2, 12 h	97	11.9	Ref[38]

[†]Nanoparticle catalyst

[‡]Single atom catalyst

[#]TOF = $mol_{\text{yield of product}}$ / (mol_{metal} • h)

[a]Substrate: R^1 = COOH, R^2 = H

[b]Substrate: R^1 = $COOCH_3$, R^2 = H

[c]Substrate: R^1 = CH_3, R^2 = H

[d]Substrate: R^1 = H, R^2 = H

[e]Substrate: R^1 = H, R^2 = H

[f]Substrate: R^1 = H, R^2 = CH_3

[g]Substrate: R^1 = H, R^2 = H

[h]Substrate: 2-furaldehyde

[i]Substrate: 2-furaldehyde

[j]Substrate: 5-hydroxymethylfurfural

[k]Substrate: R^1 = H, R^2 = H

Supplementary Methods

^{1}H NMR and ^{13}C NMR spectra were recorded at room temperature on Zhongke-Niujin 400 using $CDCl_3$, D_2O solvents. High resolution mass spectra (HRMS) were tested on Agilent 6530 Accurate-Mass Q-TOF LC/MS with ESI mode. High Performance Liquid Chromatography (HPLC) analysis for the ee values was performed on a SHIMADZU system (SHIMADZU LC-20AT pump, SHIMADZU LC-20A Absorbance Detector).

(R)-N-benzyl-1-phenylethan-1-amine (5n)

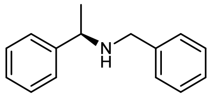

^{1}H NMR (400 MHz, $CDCl_3$) 7.35-7.21(m, 10H), 3.79 (q, J = 6.6 Hz, 1H), 3.69 – 3.53 (m, 2H), 1.59 (s, 1H), 1.34 (d, J = 6.6 Hz, 3H). **^{13}C NMR (101 MHz, $CDCl_3$)** δ 145.71, 140.79, 128.60, 128.48, 128.25, 127.05, 126.96, 126.83, 57.63, 51.79, 24.65. **HRMS (ESI)** Calcd for $C_{15}H_{17}N$ $[M+H]^+$ 212.1439; found 212.1459. **HPLC** (Daicel Chiralcel OD-H, 25 °C, n-heptane/i-PrOH = 99/1, flow rate 0.5 mL/min, λ = 210 nm). **Colorless oil.**

(S)-N-benzyl-1-phenylethan-1-amine (5o)

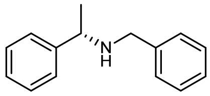

^{1}H NMR (400 MHz, $CDCl_3$) δ 7.35-7.21 (m, 10H), 3.79 (q, J = 6.6 Hz, 1H), 3.61 (q, J = 13.1 Hz, 2H), 1.60 (s, 1H), 1.35 (dd, J = 6.6, 1.0 Hz, 3H). **^{13}C NMR (101 MHz, $CDCl_3$)** δ 145.67, 140.75, 128.53, 128.42, 128.19, 126.99, 126.90, 126.77, 57.57, 51.74, 24.58. **HRMS (ESI)** Calcd for $C_{15}H_{17}N$ $[M+H]^+$ 212.1439; found 212.1446. **HPLC** (Daicel Chiralcel OD-H, 25 °C, n-heptane/i-PrOH = 99/1, flow rate 0.5 mL/min, λ = 210 nm). **Colorless oil.**

1-(4-(tert-butyl)benzyl)-4-((4-chlorophenyl)(phenyl)methyl)piperazine (5p)

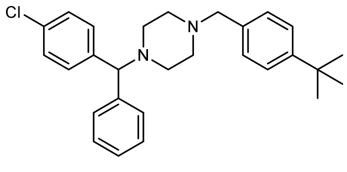

^{1}H NMR (400 MHz, $CDCl_3$) δ 7.34-7.28 (m, 6H), 7.25 - 7.15 (m, 6H), 7.15 - 7.10 (m, 1H), 4.18 (s, 1H), 3.46 (s, 2H), 2.41 (d, J = 27.7 Hz, 8H), 1.28 (s, 9H). **^{13}C NMR (101 MHz, $CDCl_3$)** δ 149.98, 142.31, 141.54, 135.02, 132.61, 129.38, 129.14, 128.73, 128.66, 128.03, 127.22, 125.17, 75.58, 62.81, 53.43, 51.94, 34.56, 31.57. **HRMS (ESI)** Calcd for $C_{28}H_{33}ClN_2$ $[M+H]^+$ 433.2411; found 433.2400. **Brown gum.**

4-(aminomethyl)benzoic acid (5q)

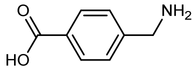

^{1}H NMR (400 MHz, D_2O) δ 7.71-7.86(m, 2H), 7.32-7.30(m,2H), 4.06(s,2H). **^{13}C NMR (101 MHz, D_2O)** 175.02, 136.95, 135.29, 129.40, 128.53, 42.71. **HRMS (ESI)** Calcd for $C_8H_{10}NO_2$ $[M+H]^+$ 152.0712; found 152.0705. **White solid.**

Supplementary References

1. Chen, F. et al. Selective catalytic hydrogenation of heteroarenes with N-graphene-modified cobalt nanoparticles (Co_3O_4-Co/NGr@α-Al_2O_3). *J. Am. Chem. Soc.* **137**, 11718-11724 (2015).

2. Huang, R. et al. Integration of metal single atoms on hierarchical porous nitrogen-doped carbon for highly efficient hydrogenation of large-sized molecules in the pharmaceutical industry. *ACS Appl. Mater. Interfaces* **12**, 17651-17658 (2020).

3. Li, J. et al. Different active sites in a bifunctional Co@N-doped graphene shells based catalyst for the oxidative dehydrogenation and hydrogenation reactions. *J. Catal.* **355**, 53-62 (2017).

4. Wei, Z. et al. Cobalt encapsulated in N-doped graphene layers: an efficient and stable catalyst for hydrogenation of quinoline compounds. *ACS Catal.* **6**, 5816-5822 (2016).

5. Sahoo, B. et al. A robust iron catalyst for the selective hydrogenation of substituted (iso)quinolones. *Chem. Sci.* **9**, 8134-8141 (2018).

6. Jiang, H. -Y., Zhang, S. -S. & Sun, B. Highly selective hydrogenation with ionic liquid stabilized nickel nanoparticles. *Catal. Lett.* **148**, 1336-1344 (2018).

7. Jagadeesh, R. V. et al. Nanoscale Fe_2O_3-based catalysts for selective hydrogenation of nitroarenes to anilines. *Science* **342**, 1073-1076 (2013).

8. Westerhaus, F. A. et al. Heterogenized cobalt oxide catalysts for nitroarene reduction by pyrolysis of molecularly defined complexes. *Nat. Chem.* **5**, 537-543 (2013).

9. Sun, X. et al. Single cobalt sites in mesoporous N-doped carbon matrix for selective catalytic hydrogenation of nitroarenes. *J. Catal.* **357**, 20-28 (2018).

10. Schwob, T. & Kempe, R. A reusable Co catalyst for the selective hydrogenation of functionalized nitroarenes and the direct synthesis of imines and benzimidazoles from nitroarenes and aldehydes. *Angew. Chem. Int. Ed.* **55**, 15175-15179 (2016).

11. Wei, Z. et al. In situ-generated Co^0-Co_3O_4/N-doped carbon nanotubes hybrids as efficient and chemoselective catalysts for hydrogenation of nitroarenes. *ACS Catal.* **5**, 4783-4789 (2015).

12. Jagadeesh, R. V. et al. Cobalt-based nanocatalysts for green oxidation and hydrogenation processes. *Nat. Protocols* **10**, 916-926 (2015).

13. Chen, J. et al. A highly active non-precious metal catalyst based on Fe-N-C@CNTs for nitroarene reduction. *RSC Adv.* **6**, 96203-96209 (2016).

14. Shi, J., Wang, Y., Du, W. & Hou, Z. Synthesis of graphene encapsulated Fe_3C in carbon nanotubes from biomass and its catalysis application. *Carbon* **99**, 330-337 (2016).

15. Xu, S., Yu, D., Liao, S., Ye, T. & Sheng, H. Nitrogen-doped carbon supported iron oxide as efficient catalysts for chemoselective hydrogenation of nitroarenes. *RSC Adv.* **6**, 96431-96435 (2016).

16. Sahoo, B. et al. Biomass-derived catalysts for selective hydrogenation of nitroarenes. *ChemSusChem* **10**, 3035-3039 (2017).

17. Wang, Y. et al. Carbon film encapsulated Fe_2O_3: an efficient catalyst for hydrogenation of nitroarenes. *Chinese. J. Catal.* **38**, 1909-1917 (2017).

18. Sun, X. et al. Metal-organic framework mediated cobalt/nitrogen-doped carbon hybrids as efficient and chemoselective catalysts for the hydrogenation of nitroarenes. *ChemCatChem* **9**, 1854-1862 (2017).

19. Zhang, F. et al. In situ mosaic strategy generated Co-based N-doped mesoporous carbon for highly selective hydrogenation of nitroaromatics. *J. Catal.* **348**, 212-222 (2017).

20. Yang, S. et al. MOF-derived cobalt phosphide/carbon nanocubes for selective hydrogenation of nitroarenes to anilines. *Chem. -Eur. J.* **24**, 4234-4238 (2018).

21. Ji, P. et al. Single-site cobalt catalysts at new $Zr_{12}(\mu_3\text{-}O)_8(\mu_3\text{-}OH)_8(\mu_2\text{-}OH)_6$ metal-organic framework nodes for highly active hydrogenation of nitroarenes, nitriles, and isocyanides. *J. Am. Chem. Soc.* **139**, 7004-7011 (2017).

22. Zheng, Y. et al. A green reduction of aromatic nitro compounds to aromatic amines over a novel Ni/SiO_2 catalyst passivated with a gas mixture. *Catal. Lett.* **124**, 268-276 (2008).

23. Hahn, G., Ewert, J. -K., Denner, C., Tilgner, D. & Kempe, R. A reusable mesoporous nickel nanocomposite catalyst for the selective hydrogenation of nitroarenes in the presence of sensitive functional groups. *ChemCatChem* **8**, 2461-2465 (2016).

24. Shu, Y. et al. Ni/Mo_2C nanowires and their carbon-coated composites as efficient catalysts for nitroarenes hydrogenation. *Appl. Surf. Sci.* **396**, 339-346 (2017).

25. Zhang, P. et al. Magnetically recoverable Ni/C catalysts with hierarchical structure and high-stability for selective hydrogenation of nitroarenes. *Phys. Chem. Chem. Phys.* **17**, 145-150 (2015).

26. Ren, Y. et al. Oxygen surface groups of activated carbon steer the chemoselective hydrogenation of substituted nitroarenes over nickel nanoparticles. *Chem. Comm.* **53**, 1969-1972 (2017).

27. Qu, Y., Yang, H., Wang, S., Chen, T. & Wang, G. Hydrogenation of nitrobenzene to aniline catalyzed by C_{60}-stabilized Ni. *Catal. Comm.* **97**, 83-87 (2017).

28. Pisiewicz, S. et al. Synthesis of nickel nanoparticles with N-doped graphene shells for catalytic reduction reactions. *ChemCatChem* **8**, 129-134 (2016).

29. Huang, H. et al. Nitrogen-doped graphene-activated metallic nanoparticle-incorporated ordered mesoporous carbon nanocomposites for the hydrogenation of nitroarenes. *RSC Adv.* **8**, 8898-8909 (2018).

30. Ryabchuk, P. et al. Intermetallic nickel silicide nanocatalyst-A non-noble metal-based general hydrogenation catalyst. *Sci. Adv.* **4**, eaat0761 (2018).

31. Jagadeesh, R. V. et al. MOF-derived cobalt nanoparticles catalyze a general synthesis of amines. *Science* **358**, 326-332 (2017).

32. Murugesan, K., Beller, M. & Jagadeesh, R. V. Reusable nickel nanoparticles-catalyzed reductive amination for selective synthesis of primary amines. *Angew. Chem. Int. Ed.* **58**, 5064-5068 (2019).

33. Hahn, G., Kunnas, P., de Jonge, N. & Kempe, R. General synthesis of primary amines via reductive amination employing a reusable nickel catalyst. *Nat. Catal.* **2**, 71-77 (2019).

34. Bäumler, C., Bauer, C. & Kempe, R. The synthesis of primary amines through reductive amination employing an iron catalyst. *ChemSusChem* **13,** 3110-3114 (2020).

35. Liu, X., Wang, Y., Jin, S., Li, X. & Zhang, Z. High performance of nitrogen-doped carbon-supported cobalt catalyst for the mild and selective synthesis of primary amines. *Arab. J. Chem.* **13**, 4916-4925 (2020).

36. Zhou, K. et al. Selective synthesis of furfurylamine by reductive amination of furfural over Raney cobalt. *ChemCatChem* **11**, 5562-5569 (2019).

37. Yuan, H. et al. Reductive amination of furanic aldehydes in aqueous solution over versatile Ni_yAlO_x catalysts. *ACS Omega* **4**, 2510-2516 (2019).

38. Yuan, Z., Liu, B., Zhou, P., Zhang, Z. & Chi, Q. Preparation of nitrogen-doped carbon supported cobalt catalysts and its application in the reductive amination. *J. Catal.* **370**, 347-356 (2019).
